# Characterization of a putative sensory [FeFe]-hydrogenase provides new insight into the role of the active site architecture†

**DOI:** 10.1039/d0sc03319g

**Published:** 2020-09-21

**Authors:** Henrik Land, Alina Sekretareva, Ping Huang, Holly J. Redman, Brigitta Németh, Nakia Polidori, Lívia S. Mészáros, Moritz Senger, Sven T. Stripp, Gustav Berggren

**Affiliations:** Molecular Biomimetics, Department of Chemistry, Ångström Laboratory, Uppsala University Box 523 SE-75120 Uppsala Sweden gustav.berggren@kemi.uu.se; Physical Chemistry, Department of Chemistry, Ångström Laboratory, Uppsala University Box 523 SE-75120 Uppsala Sweden; Bioinorganic Spectroscopy, Department of Physics, Freie Universität Berlin Arnimallee 14 DE-14195 Berlin Germany sven.stripp@fu-berlin.de

## Abstract

[FeFe]-hydrogenases are known for their high rates of hydrogen turnover, and are intensively studied in the context of biotechnological applications. Evolution has generated a plethora of different subclasses with widely different characteristics. The M2e subclass is phylogenetically distinct from previously characterized members of this enzyme family and its biological role is unknown. It features significant differences in domain- and active site architecture, and is most closely related to the putative sensory [FeFe]-hydrogenases. Here we report the first comprehensive biochemical and spectroscopical characterization of an M2e enzyme, derived from *Thermoanaerobacter mathranii*. As compared to other [FeFe]-hydrogenases characterized to-date, this enzyme displays an increased H_2_ affinity, higher activation enthalpies for H^+^/H_2_ interconversion, and unusual reactivity towards known hydrogenase inhibitors. These properties are related to differences in active site architecture between the M2e [FeFe]-hydrogenase and “prototypical” [FeFe]-hydrogenases. Thus, this study provides new insight into the role of this subclass in hydrogen metabolism and the influence of the active site pocket on the chemistry of the H-cluster.

## Abstract 2

Characterization of a group D putative sensory [FeFe]-hydrogenase reveals how the active site can be tuned to decrease CO inhibition and increase stability of a reduced H-cluster while retaining the ability to catalyze H^+^/H_2_ interconversion.

## Graphical abstract

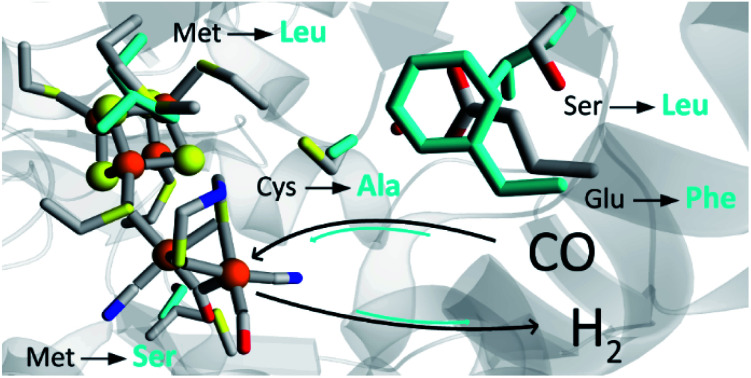

## Introduction

Hydrogenase enzymes play a central role in hydrogen metabolism, where they catalyze the interconversion between protons and molecular hydrogen (H_2_). The [FeFe]-hydrogenases are generally considered the most active, operating close to the thermodynamic limit with reported H_2_ production rates exceeding 9000 s^−1^.^1,2^ Consequently, they have been intensively studied, both for their biotechnological potential and as a model system for the design of synthetic catalysts.^3,4^ Phylogenetically, [FeFe]-hydrogenases can be broadly divided into four main groups, denoted group A, B, C, and D, which in turn contain numerous subclasses.^5–9^ Considering the well-conserved nature of the auxiliary proteins involved in cofactor assembly (HydEFG),^6^ they all arguably share a dependence on the same hexanuclear iron cofactor, the “H-cluster”. This biologically unique cofactor consists of a canonical [4Fe–4S] cluster ([4Fe–4S]_H_) connected to a low valent dinuclear iron complex ([2Fe]_H_).^10–13^ The [2Fe]_H_ subsite is coordinated by CO and CN^−^ ligands, and bridged by an azadithiolate ligand (adt = ^−^SCH_2_NHCH_2_S^−^). The overwhelming majority of biochemically characterized [FeFe]-hydrogenases belong to group A, with a primary focus on the “prototypical” [FeFe]-hydrogenases, *e.g.*, *Cr* HydA1 from *Chlamydomonas reinhardtii*,^14,15^*Dd* HydAB from *Desulfovibrio desulfuricans*,^11,16,17^*Cp*I from *Clostridium pasteurianum*,^10,18^ as well as the multimeric electron bifurcating [FeFe]-hydrogenase from *Thermotoga maritima*.^18–20^ Studies of these enzymes form the foundation for our understanding of [FeFe]-hydrogenase biochemistry. Spectroscopy has identified numerous redox and protonation states of the H-cluster, around which various mechanistic proposals have been put forth.^21–24^ In short, the active-ready resting state (H_ox_) features a mixed valence Fe(ii)Fe(i) form of the [2Fe]_H_ subsite and an oxidized [4Fe–4S]_H_ cluster (2+). One-electron reduction results in either the H_red′_ or H_red_ state, where H_red′_ features a reduced [4Fe–4S]_H_ cluster while H_red_ features a reduced and protonated [2Fe]_H_ subsite.^25^ Further reduction results in the formation of the so-called H_hyd_ state featuring a terminal hydride on the [2Fe]_H_ subsite.^26–28^ Protonation of H_hyd_ results in H_2_ release, potentially proceeding *via* a discrete intermediate (H_hyd_H^+^),^27,29^ and returns the H-cluster to the H_ox_ state. Additionally, CO can reversibly bind to the H-cluster, giving rise to the inhibited H_ox_-CO and H_red′_-CO states.^30^

Considering the diverse nature of [FeFe]-hydrogenase, both with regards to structure as well as function, it is clear that characterization of representative examples from other subclasses is necessary to complete our understanding of this enzyme family and H-cluster chemistry. It has repeatedly been shown that [FeFe]-hydrogenases can operate at minimal over-potentials, albeit specific enzymes generally display a bias for either H^+^ reduction or H_2_ oxidation.^31–34^ Indeed, even in the relatively narrow selection of enzymes studied to-date significant differences in catalytic rates, stability of different H-cluster states and reactivity towards inhibitors (*e.g.*, CO and O_2_) have been observed.^22,31,35–39^ On a fundamental level, further insight into subclass-specific reactivities is critical for our understanding of hydrogen metabolism, and elucidating the interplay between the H-cluster and the protein. It will also serve to strengthen efforts related to biotechnological energy applications and potentially facilitate the development of selective antibiotics.^8,9^

**Fig. 1:**
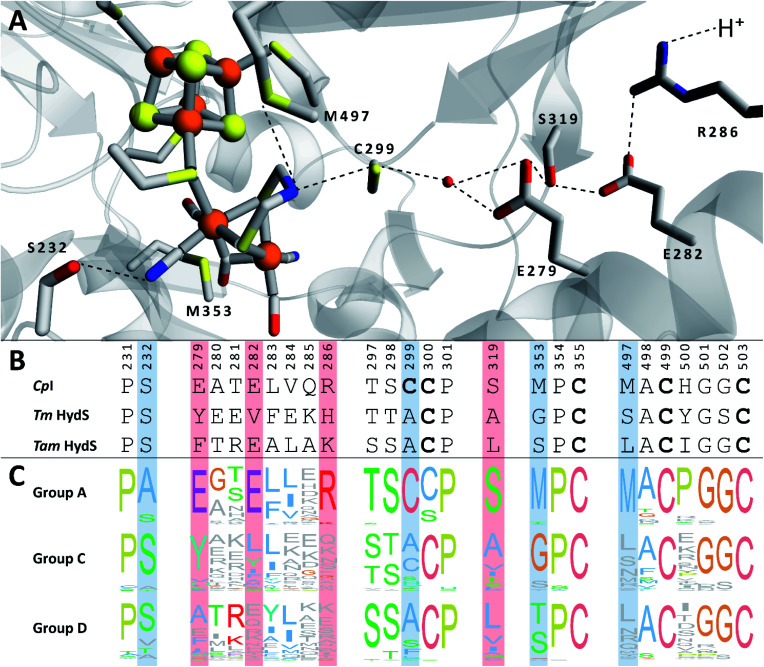
(A) Structural view of the active site and proton transfer pathway of a prototypical [FeFe]-hydrogenase. Structure and numbering based on *Cp*I (PDB ID: 4XDC). Shown amino acid residues are either involved in interactions with the H-cluster or in the proton transfer pathway and show large variations between groups A, C and D. Potential interactions are shown with dashed lines. (B) Amino acid sequence comparison of *Cp*I, *Tm* HydS and *Tam* HydS (*Cp*I numbering) based on a ClustalΩ sequence alignment^80^ of sequences retrieved from Greening *et al.* 2016 (ref. 7) and homology modeling. H-cluster interacting cysteine residues are highlighted in bold. (C) Normalized consensus logos of [FeFe]-hydrogenase groups A, C and D generated in Jalview using the sequence alignment in (B). Coloring is based on the Clustal X color scheme. Amino acid residues involved in H-cluster interaction and proton transfer that show variation between the groups are highlighted in blue and red, respectively.

We recently reported the whole-cell characterization of an [FeFe]-hydrogenase from the thermophilic firmicute *Thermoanaerobacter mathranii* in *E. coli*.^40^ The enzyme belongs to the hitherto uncharacterised M2e subclass, which displays a number of well-conserved differences in amino acid sequence as compared to the prototypical group A hydrogenases; namely in the active site cavity and the proton transfer pathway (Fig. 1).^41^ The M2e subclass has been proposed to form a distinct group of [FeFe]-hydrogenases, group D, and their physiological function is unknown.^5^ Still, it is phylogenetically most closely related to the M2f subclass of group C, which has been identified as “putative sensory” and includes the recently characterized *Tm* HydS enzyme from *Thermotoga maritima*.^5,7,8,38^ In addition to the H-domain, which harbors the H-cluster, both subclasses feature an N-terminal domain with two [4Fe–4S] cluster-binding motifs as well as a C-terminal domain with a four-cysteine motif (Cx_2_Cx_4_Cx_16_C) indicative of iron–sulfur (FeS) cluster binding. Additionally, the highly conserved cysteine residue initiating the proton transfer pathway from the adt amine in group A [FeFe]-hydrogenase is not conserved in the M2e and M2f subclasses (C299, *Cp*I numbering is used throughout the text unless otherwise noted). Indeed, all residues that have previously been shown to be crucial for proton transfer in group A are missing in these subclasses (Fig. 1).^42–44^ Moreover, the genes encoding M2e and M2f enzymes are often located on the same operon as group A hydrogenases.^5^ In light of these similarities, we have now denoted the enzyme as *Tam* HydS (previously *Tam* HydA^40^). However, there are key differences between the two subclasses. In contrast to the M2f enzymes, the M2e subclass does not feature a Per-Arnt-Sim (PAS) domain, which was used to propose the sensing function of group C, as it is usually involved in the regulation of histidine kinases associated with signal transduction.^5^ In addition, the active site architecture displays distinct differences between the two subclasses (Fig. 1). The two methionine residues framing the diiron site, M497 and M353, are exchanged against serine and glycine in *Tm* HydS; and leucine and serine, respectively, in *Tam* HydS. These methionines are considered critical for modulating the reactivity of prototypical [FeFe]-hydrogenases,^31,41^ and it is noteworthy that the exchange of M353 to a hydroxyl donor (serine or threonine) appears to be a well-conserved property of M2e enzymes. On the other hand, M497 shows a low level of conservation, both for group C and D enzymes.

Herein, we report the first detailed characterization of an M2e enzyme, and by extension a group D [FeFe]-hydrogenase. The aforementioned *Tam* HydS enzyme has been isolated following heterologous expression, and characterized through electron paramagnetic resonance (EPR) spectroscopy, attenuated total reflection Fourier-transform infrared (ATR FTIR) spectroscopy, and protein film electrochemistry (PFE). Despite lacking several amino acid residues considered critical for the activity of prototypical [FeFe]-hydrogenase, *Tam* HydS shows reversible H^+^/H_2_ interconversion close to the thermodynamic potential, with a slight bias for H^+^ reduction. These findings show that the active site pocket can be significantly altered while still retaining the catalytic function of the H-cluster. However, in contrast to previously characterized [FeFe]-hydrogenases, an over-potential is observed at low temperatures; and the overall catalytic rates are low. Moreover, the M2e enzyme displays significant differences in its reactivity towards CO and O_2_, as compared to previously characterized [FeFe]-hydrogenases. We propose that the catalytic rates are influenced by intramolecular proton transfer. The study, furthermore, highlights the importance of the active site pocket in modulating the reactivity towards known inhibitors, and the stability of specific H-cluster states. Based on the aforementioned properties of *Tam* HydS which resemble those of known regulatory [NiFe]-hydrogenases, in combination with analysis on a genome level, we hypothesize that group D [FeFe]-hydrogenases serve a sensory rather than catalytic function.

## Results and discussion

### Isolation and characterization of *apo-Tam* HydS

Aerobic heterologous expression of *Tam* HydS in *E. coli*, in the absence of the HydEFG maturation enzymes, has been shown to result in soluble *apo*-protein that could be activated *in vivo* using [2Fe]_H_ subsite mimics.^40^ In this study, anaerobic isolation of *apo-Tam* HydS resulted in a purified protein (Fig. S1†) containing 13.9 ± 0.85 Fe/protein. Additional reconstitution of the FeS clusters resulted in a final Fe content of 16.1 ± 0.29 Fe/protein, as expected from the incorporation of four [4Fe–4S] clusters. Isolation of *apo-Tam* HydS also under aerobic conditions provided a protein with an Fe content of 10.6 ± 0.06 Fe/protein.

**Fig. 2:**
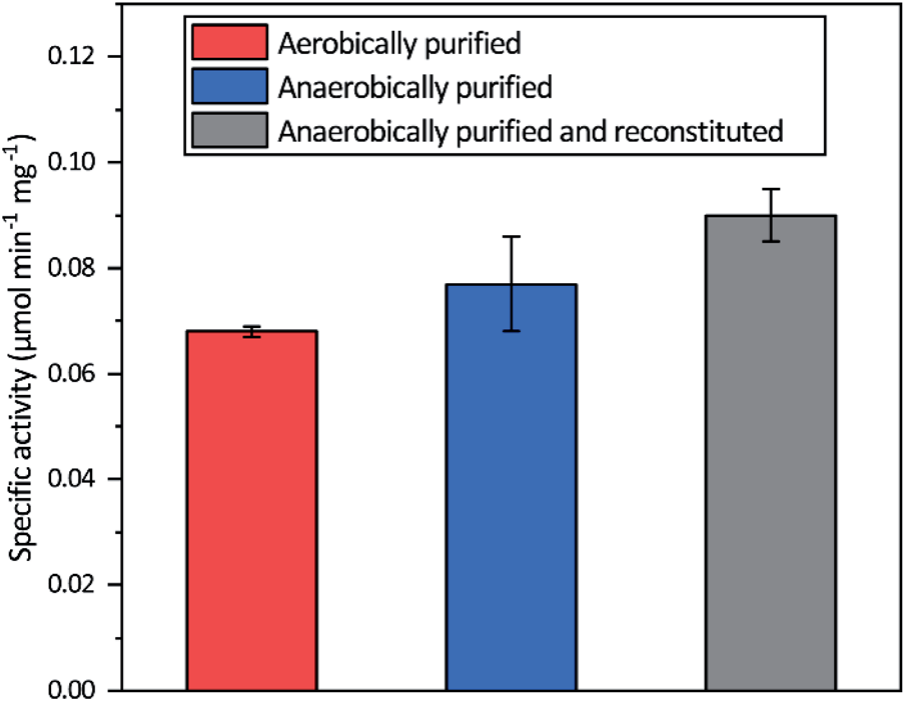
Specific H_2_ production activities of *Tam* HydS isolated under different conditions and activated *in vitro* with [2Fe]^adt^. The Fe content of the different preparations prior to insertion of [2Fe]^adt^ were 10.9 ± 0.06 (aerobic purification), 13.9 ± 0.85 (anaerobic purification) and 16.1 ± 0.29 (reconstituted) Fe/protein. Reactions were performed in sodium phosphate buffer (100 mM, pH 6.8) with Triton X-100 (1% v/v), methyl viologen (10 mM) and sodium dithionite (100 mM).

All three different purified forms of *apo-Tam* HydS displayed hydrogenase activity in *in vitro* sodium dithionite/methyl viologen (NaDT/MV^2+^) enzymatic assays, following anaerobic incubation with the synthetic [2Fe]_H_ analogue [Fe_2_(adt)(CO)_4_(CN)_2_]^2−^ ([2Fe]^adt^). Activation of aerobically and anaerobically purified *apo-Tam* HydS samples resulted in H_2_ production activities of 0.068 ± 0.001 and 0.077 ± 0.009 μmol H_2_ per min per mg, respectively. A further increase in the H_2_ production activity, to 0.090 ± 0.005 μmol H_2_ per min per mg was observed after activation of the reconstituted samples (Fig. 2).

**Fig. 3:**
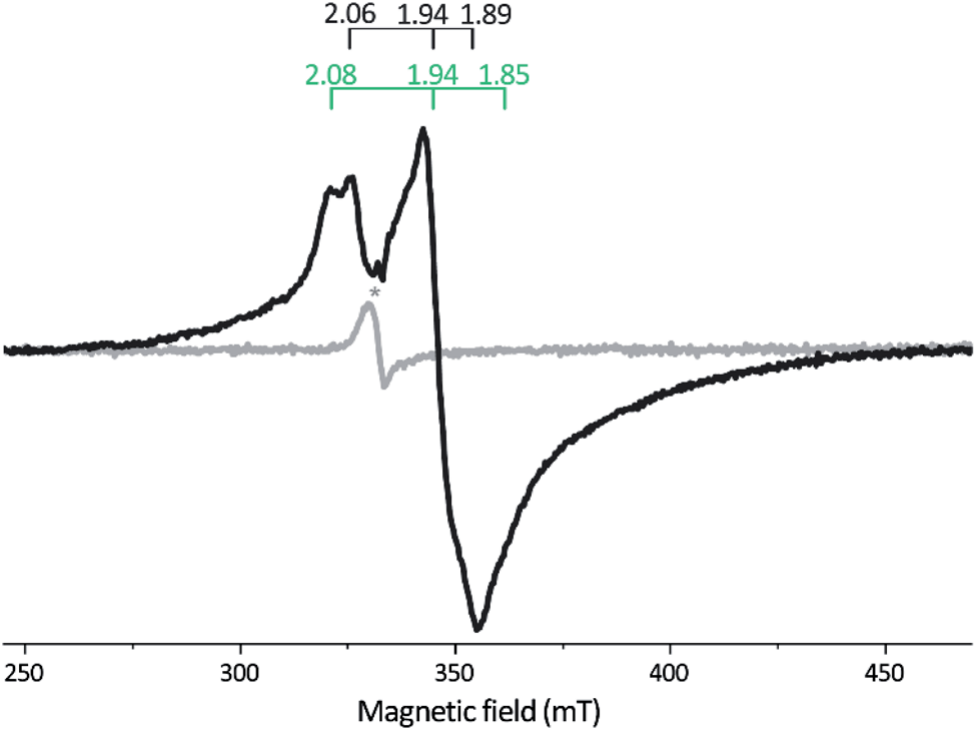
X-band EPR spectra of reconstituted *apo-Tam* HydS (50 μM). Gray spectrum: as prepared; black spectrum: NaDT reduced. The observed rhombic signal in the reduced sample is typical for [4Fe–4S]^+^ clusters. The *g*-tensors are indicated for the two contributing components, identified through comparison to NaDT reduced *holo-Tam* HydS (see Fig. S3†). The weak signal marked with * appearing at *g* = 2.02 is attributed to a trace amount of [3Fe–4S]^2+^. EPR settings: *T* = 17 K; modulation frequency 100 kHz, amplitude 15 G; microwave frequency 9.4 GHz, power 8 mW.

X-Band EPR spectra recorded on samples of as-isolated and reconstituted *apo-Tam* HydS are essentially silent, indicating that all FeS-clusters are present at a [4Fe–4S]^2+^ oxidation state with spin *s* = 0, thus diamagnetic. Reduction of the reconstituted enzyme by NaDT at pH 8 brought the sample to an EPR active state, displaying a broad rhombic EPR spectrum near the *g* ∼ 2 region, typical of *s* = 1/2 [4Fe–4S]^+^ species (Fig. 3). Spin quantification resulted in two spin centers per protein, suggesting that two of the [4Fe–4S] clusters are susceptible to NaDT reduction.

The iron quantification supports the presence of four [4Fe–4S] clusters in the reconstituted protein, including the active-site [4Fe–4S]_H_ cluster. This assignment is further supported by EPR spectroscopy. Albeit only two of the clusters were clearly discernable, no signal attributable to [2Fe–2S] clusters was observed upon reduction, and only traces of a [3Fe–4S] cluster signal were present in the non-reduced samples.^45,46^ Similar results were recently reported for the putative sensory M2f enzyme *Tm* HydS, and it appears to be a shared trait between the two subclasses.^38^ This FeS cluster composition is also supported by the linear increase in activity as iron content is increased up to 16 Fe/protein. Although the H_2_ production activity of *Tam* HydS, observed in *in vitro* assays, is low compared to most known prototypical [FeFe]-hydrogenases, it is similar to *Tm* HydS.^38^ As the two N-terminal [4Fe–4S] clusters are well conserved across numerous [FeFe]-hydrogenases, including the aforementioned *Dd* HydAB as well as the M2 enzyme from *Megasphaera elsdenii* this arguably represents the catalytic electron transfer pathway also in the M2e enzymes.^21,32,39^ In contrast, the biochemical and physiological role of the [4Fe–4S] cluster coordinated by the C-terminal Cx_2_Cx_4_Cx_16_C motif is unknown. As it is located between the H-domain and the PAS domain in M2f [FeFe]-hydrogenases, it is likely to be involved in a signaling process.

### EPR characterization of *holo-Tam Hyd*S

The *in vitro* enzymatic assays revealed that treating *apo-Tam* HydS with [2Fe]^adt^ resulted in spontaneous formation of *holo-Tam* HydS on a minute time-scale, similar to what was previously observed under whole-cell conditions.^40^ In the absence of reductant (NaDT), this treatment is expected to yield an oxidized form of the H-cluster, either the H_ox_ state or the H_ox_-CO state.^13,47–49^ Both states are paramagnetic and best described as [4Fe–4S]_H_^2+^–[2Fe(ii,i)] (*s* = 1/2) species, thus EPR spectroscopy can be employed to monitor H-cluster assembly. It is well established that the EPR spectra of H_ox_ and H_ox_-CO generally display rhombic and axial signals, respectively, with small anisotropy and spin transitions at *g* ≈ 2.^22^

**Fig. 4:**
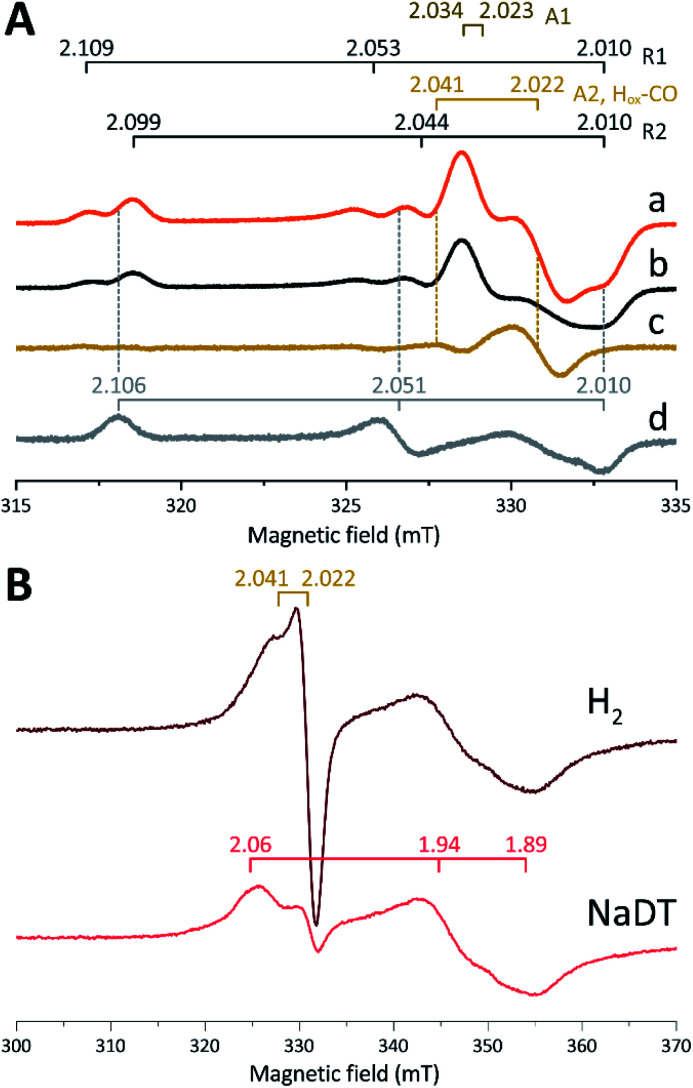
(A) Signals observed for *holo-Tam* HydS and attributed to the H-cluster, (a) *holo-Tam* HydS activated with [2Fe]^adt^ as isolated; (b) *holo-Tam* HydS flushed by N_2_ to remove H_ox_-CO contributions (contains components R1, R2, A1 and A2); (c) difference spectra obtained from subtraction of spectrum (b) from (a) revealing an axial signal attributed to H_ox_-CO (“component A2, H_ox_-CO”); (d) *Tam* HydS activated with [2Fe]^pdt^ (pdt-*Tam* HydS, as isolated). EPR settings: *T* 21 K; modulation frequency 100 kHz, amplitude 10 G; microwave frequency 9.4 GHz, power 16 μW. (B) Reduction of *holo-Tam* HydS activated with [2Fe]^adt^ by H_2_ and NaDT resulting in disappearance of the H-cluster signals and appearance of a rhombic signal attributed to a [4Fe–4S]^+^ cluster. EPR settings: *T* 10 K; modulation frequency 100 kHz, amplitude 15 G; microwave frequency 9.4 GHz, power 80 μW. *g*-values indicated in the figure on horizontal bars, for details see main text.

Expecting a mixture of H_ox_ and H_ox_-CO, the X-band EPR spectra collected on solution samples of as-prepared *holo-Tam* HydS, generated under an inert argon atmosphere, displayed a surprisingly complex pattern. As seen in Fig. 4A (spectrum a), at least seven features were resolved. The spectrum displayed characteristics suggesting the presence of H_ox_ and H_ox_-CO but the unusual complexity of the spectrum shows that more than two species contribute to the overall spectrum. Spin quantification of a representative spectrum resulted in 0.64 spin per protein, indicating that a fraction of the enzyme also resided in an EPR silent state, assigned by FTIR spectroscopy to the H_red_ state (see below). The relaxation behavior of the signal(s) for *holo-Tam* HydS was estimated by monitoring the dominant *g* ≈ 2.04–2.02 feature (Fig. S4†). All components indicated in the spectrum followed similar saturation trends, and displayed low *P*_1/2_ values (73 μW and 1.15 mW at 15 and 21 K, respectively). This suggests that spin relaxation is a slow process, most likely due to isolation of the H-cluster from the lattice.

Flushing of *holo-Tam* HydS solutions with N_2_ prior to freezing resulted in approx. 50% decrease of total spin density, reflected in a minor decrease in amplitude of the low-field features (*g* ≈ 2.1) while a pseudo-axial component represented by a feature at *g* = 2.022 was almost completely lost (Fig. 4A, spectrum b). Subtraction of the spectrum obtained following N_2_ flushing (spectrum b) from the spectrum of the as-prepared sample (spectrum a) provided a “pure” pseudo-axial spectrum (Fig. 4A, spectrum c) with *g*_‖_ = 2.041 and *g*_⊥_ = 2.022. This signal is assigned to the H_ox_-CO state (Fig. 4A, component “A2, H_ox_-CO”). Similarly, the H_ox_-CO component was diminished in samples of *holo-Tam* HydS isolated following H-cluster assembly in the presence of NaDT (Fig. S5†). The residual spectrum (spectrum b) appears to feature two sets of rhombic signals (components R1 and R2), in combination with an additional narrow axial signal (component A1). The *g*_‖_ = 2.034 tensor of the latter signal is readily apparent while the g_⊥_ tensor is tentatively assigned to 2.023 from simulations (indicated in dark brown in Fig. 4A, see Fig. S5† for simulation details). With regards to the rhombic signals, the *g*-values are 2.109 and 2.099 for the two features at the lower field wing, 2.053 and 2.044 in the centre region and 2.010 in the high field wing. The overall spectral shape was highly similar between biological repeats prepared at pH 8. The relative signal amplitudes observed in spectrum (b) were also retained at pH 5 (Fig. S5†) but this acidification resulted in a 2–3 gauss downshift of one set of the rhombic EPR signals, which facilitated separation into two sets of separate *g*-tensors (*g*_*zyx*_ = 2.109, 2.053, 2.010, “component R1”; and *g*_*zyx*_ = 2.099, 2.044, 2.010, “component R2”; Fig. 4A). It should be noted that the assignment of *g*_*x*_ = 2.010 is speculative due to its overlap with the adjacent axial signal.

To further clarify the EPR spectrum observed for *Tam* HydS, the *holo*-enzyme was generated using the modified cofactor [Fe_2_(pdt)(CO)_4_(CN)_2_]^2−^ ([2Fe]^pdt^, pdt = ^−^SCH_2_CH_2_CH_2_S^−^; pdt-*Tam* HydS). It has been shown for both group A and C [FeFe]-hydrogenases that replacing the amine-bridgehead of the adt ligand with a methylene group destabilizes the H_ox_-CO state.^13,30,38,50,51^ Thus, analogous samples where [2Fe]^pdt^ replaced [2Fe]^adt^ were examined by EPR under the same recording conditions. The obtained spectrum showed a rhombic anisotropy with *g*-values of 2.106, 2.051 and 2.010 (Fig. 4A, spectrum d). This signal is attributed to the formation of a single pure H_ox_ state, in good agreement with FTIR spectroscopy (Fig. S6†), as well as earlier studies of *Tam* HydS under whole-cell conditions.^40^ A comparison between *holo-Tam* HydS generated with [2Fe]^adt^ (Fig. 4A, spectra a and b) and [2Fe]^pdt^ (Fig. 4A, spectrum d) reveals that the rhombic features observed in spectra (a) and (b) display a significant overlap with the signal observed for pdt-*Tam* HydS (indicated with dashed lines in Fig. 4A). Consequently, the rhombic components R1 and R2 are assigned to two distinct H_ox_-like species. The non-overlapping features of the spectrum correspond to the pseudo-axial components (A1 and A2), in agreement with the assignment of A2 to the H_ox_-CO state.

Reduction of *holo-Tam* HydS with NaDT resulted in disappearance of the aforementioned H-cluster signals with concomitant appearance of a broader rhombic EPR spectrum (Fig. 4B, spectrum NaDT). The loss of the H_ox_-CO and H_ox_-like signals is attributed to a one-electron reduction of the H-cluster to the diamagnetic H_red_ state (see below). The new signal partially resembles that observed for reduced *apo-Tam* HydS (Fig. 3), and spin quantification showed one spin per protein. Thus, one [4Fe–4S] cluster, with an EPR signature of *g*_*zyx*_ = 2.06, 1.94 and 1.89, is susceptible to NaDT reduction in *holo-Tam* HydS. Subtraction of the *holo-Tam* HydS signal from that of *apo-Tam* HydS revealed another broad rhombic EPR signal (*g*_*zyx*_ = 2.08, 1.94 and 1.85, see Fig. S3,† green spectrum). As this signal was present in the *apo*-protein but lost upon H-cluster formation, it is tentatively attributed to the [4Fe–4S]_H_ cluster of *apo-Tam* HydS. Reduction of *holo-Tam* HydS with H_2_ provided a similar result compared to reduction with NaDT, although a larger fraction of the H_ox_-CO state remained (Fig. 4B, spectrum H_2_).

In summary, the combined EPR data from as-prepared and gas-flushed solution samples reveal an unusually complex mixture of oxidized states. Still, three of the contributing species can be assigned with relatively high certainty. Comparison of as-prepared and N_2_ flushed samples show that a standard H_ox_-CO species can form also in *holo-Tam* HydS. Conversely, the “split” H_ox_-like signal observed in *holo-Tam* HydS is suggestive of the formation of two distinguishable H_ox_-like states. Based on FTIR spectroscopy (see below), R2 is attributed to a state highly similar to the well-known H_ox_ state of prototypical [FeFe]-hydrogenases (*g*_*zyx*_ = 2.099, 2.044, 2.010), and the second rhombic EPR signal, R1, to a state similar to H_ox_H (*g*_*zyx*_ = 2.109, 2.053, 2.010). A similar downshift of the H_ox_-signal upon formation of H_ox_H has recently been reported for *Cr* HydA1.^9^ As comparing spectra of samples prepared at mildly basic and acidic conditions did not reveal significant changes in the relative amplitudes of the rhombic signals their interconversion appears to be more complicated than an acid–base equilibrium. The structural details of this H_ox_H-like state in *Tam* HydS remains to be fully elucidated. Still, both appear catalytically competent, as they were both lost upon exposure to H_2_.

### FTIR characterization of *holo-Tam* HydS

The H-cluster of *holo-Tam* HydS was further investigated using ATR FTIR spectroscopy at different pH values and in the presence of H_2_, N_2_, CO, or O_2_. The enzyme adopted H_red_ as a semi-stable resting state under our experimental conditions (20 °C, N_2_ atmosphere with approx. 1% H_2_, hydrated protein films at pH 7). A quantitative enrichment of H_ox_ was achieved only after 20–30 h of continuous purging with pure N_2_. In contrast to what is generally reported for prototypical [FeFe]-hydrogenases,^52^ the H_ox_-state (≈65%) accumulated together with a small fraction (≈25%) of an alternative state displaying an H_ox_-like spectrum at higher frequencies (Fig. S7†). This hypsochromically-shifted signature is attributed to an H_ox_H-like state, albeit this state is generally not observed at neutral pH in prototypical [FeFe]-hydrogenases. This mixture of H_ox_-like states and their relative ratio is in agreement with the observation of two rhombic EPR signals under similar conditions (Fig. 4). The H_red_ to H_ox_ transition was further analyzed by ATR FTIR spectro-electrochemistry, revealing two unusual properties of the *Tam* HydS enzyme (Fig. S8†). The reaction displayed a significant over-potential requirement, and while the quasi-reversible nature of the process prevented an exact assignment of the H_ox_/H_red_ reduction potential it was clearly shifted in an anodic direction as compared to previously studied prototypical [FeFe] hydrogenase, with *E*_m_s of ≈−350 to −450 mVs reported for *Cr* HydA1 and *Dd* HydAB.^39,53,54^ During the reductive scan, H_ox_/H_red_ interconversion in *Tam* HydS was observed at *E*_m_ ≈ −300 mV *vs.* SHE, while the re-oxidation did not occur until a potential of approx. +200 mV was applied (at pH 8). No intermediates were observed in the process. Thus, H_red_ appears to be both kinetically and thermodynamically stabilized in *Tam* HydS. A relatively anodic H_ox_/H_red_ midpoint potential has been reported also for the putative sensory Group C hydrogenase *Tm* HydS.^38^

**Fig. 5:**
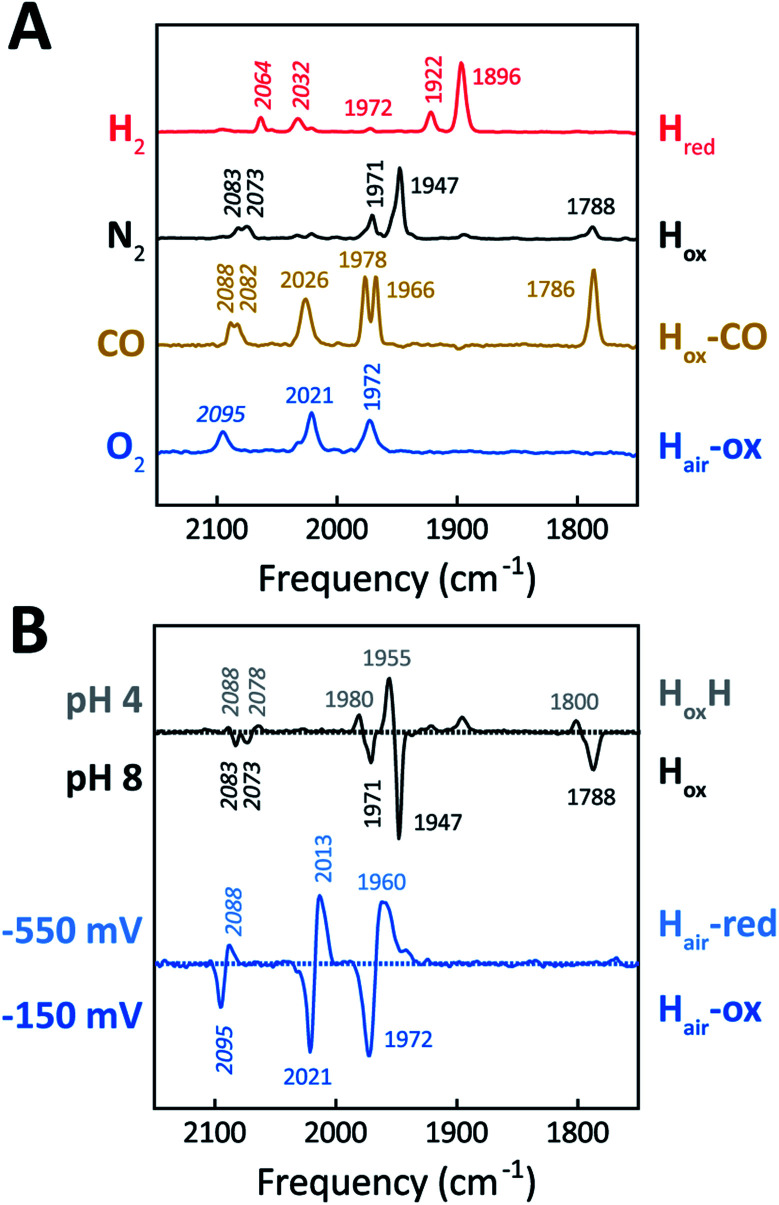
ATR FTIR characterization of *holo-Tam* HydS. All data recorded at RT. (A) Infrared signature of the H-cluster in *Tam* HydS in the presence of H_2_ (red, H_red_), N_2_ (black, H_ox_ and H_ox_H), CO (brown, H_ox_-CO), and after reaction with O_2_ (blue, H_air_). (B) The upper difference spectrum shows accumulation of H_ox_H (positive bands, grey, pH 4) over H_ox_ (negative bands, black, pH 8) under N_2_. Note: accumulation of H_ox_H also required NaDT addition. In the lower difference spectrum, FTIR spectro-electrochemistry was used to accumulate H_air_-red (positive bands, light blue, −550 mV *vs.* SHE) over H_air_-ox (negative bands, blue, −150 mV).

Fig. 5A shows the IR signatures of H_ox_, H_ox_-CO, and H_red_ observed for *Tam* HydS. The assignment of the spectra to specific H-cluster states was facilitated by their overall similarities to spectra previously reported for prototypical [FeFe]-hydrogenases. Still, the frequencies of the terminal CO/CN^−^ ligands are upshifted in comparison to *Cr* HydA1 and *Dd* HydAB and closer to *Cp*I and *Ca*I from *C. acetobutylicum*.^31,37,52,55^ The high-frequency CO band of H_ox_-CO (2026 cm^−1^) indicates a constrained geometry.^51^ In contrast to these upshifts, the *μ*CO band of H_ox_ (1788 cm^−1^) and H_ox_-CO (1786 cm^−1^) was found at lower frequencies than typically observed. These latter differences, as compared to group A and C [FeFe]-hydrogenases, are likely attributable to the M393S variation in *Tam* HydS as this residue is in close contact with the bridging *μ*CO ligand. A distinct feature in the *μ*CO region for the H_red_ state could not be discerned. At low pH and high concentrations of NaDT, accumulation of H_ox_H over H_ox_ was achieved (Fig. 5B), although the protein film never fully converted into H_ox_H (Fig. S7†). Note that the signature of H_ox_H at low pH is in excellent agreement with the hypsochromically shifted H_ox_-like spectrum observed at pH 8. In contrast to what has been reported for prototypical [FeFe]-hydrogenases, no accumulation of H_hyd_ was observed, *e.g.*, when low pH samples were exposed to H_2_.^56^ Moreover, H_sred_ and H_red′_ were never detected. Table 1 summarizes the IR signature of all observed H-cluster states.

**Table 1:** Vibrational frequencies observed for the CN^−^ and CO ligands

	CN^−^ (cm^−1^)		CO (cm^−1^)			
H_red_	2064	2032	1972	1922	1896	
H_ox_	2083	2073		1971	1947	1788
H_ox_H	2088	2078		1980	1955	1800
H_ox_-CO	2088	2082	2026	1978	1966	1786
H_air_-ox	2095		2021	1972		
H_air_-red	2088		2013	1960		

**Fig. 6:**
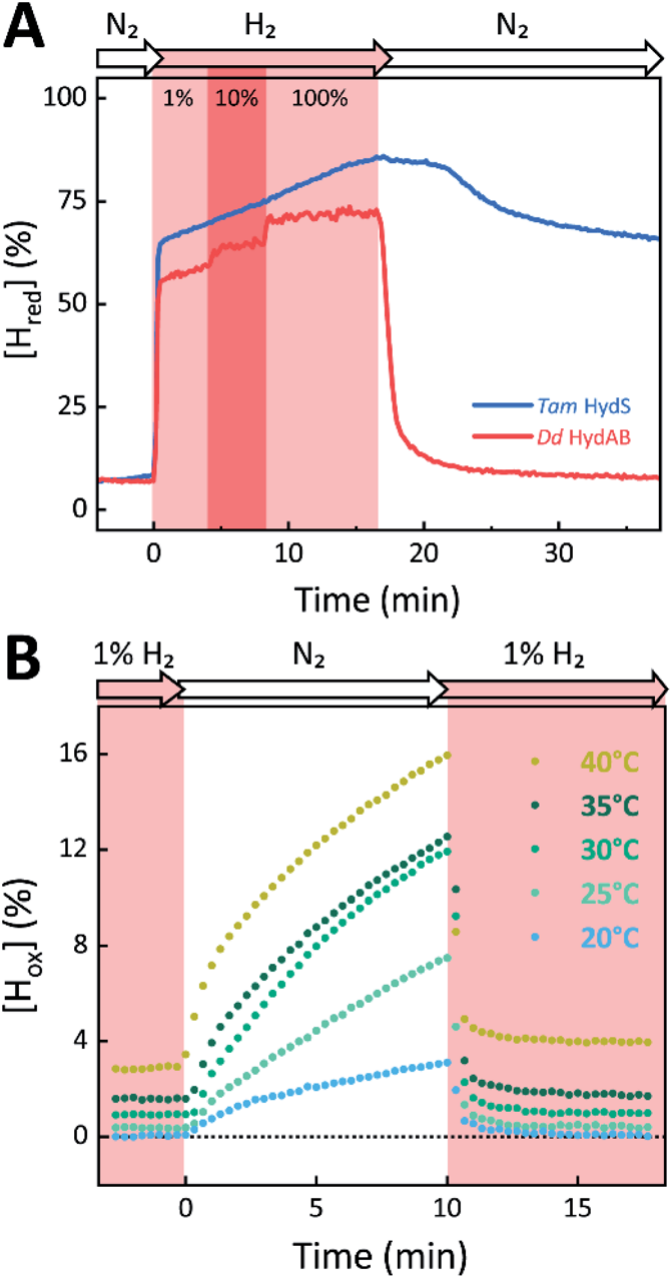
The reactivity of *Tam* HydS and prototypical [FeFe]-hydrogenase (*Dd* HydAB) towards H_2_ (A) and temperature dependence of the auto-oxidation activity (B) monitored by time-resolved ATR FTIR spectroscopy. (A) Kinetic traces of H_red_ for *Tam* HydS (blue) and *Dd* HydAB (red) that show the reaction with different concentrations of H_2_ (RT). Note the persistence of H_red_ in the absence of H_2_ for *Tam* HydS. (B) Change of [H_ox_] as a function of gas, time, and temperature. Representative data set for *Tam* HydS, recorded at pH 7. The equilibrium under 1% H_2_ is slightly shifted in favour of H_ox_ over H_red_ as temperature is increased, and so does rate of [H_ox_] formation as the atmosphere is changed to pure N_2_.

The absorbance spectra of the H-cluster states identified in Fig. 5 were fitted and used to describe the interconversion reactions as a function of gas composition and time. For comparative purposes, analogous experiments were performed with the prototypical [FeFe]-hydrogenases *Dd* HydAB or *Cr* HydA1. Fig. 6A depicts the rapid conversion of H_ox_ into H_red_ for *Tam* HydS and *Dd* HydAB at 1%, 10%, and 100% H_2_ over N_2_. *Dd* HydAB was chosen for comparison because it shows a similar, albeit not identical, composition of reduced H-cluster states under H_2_ (Fig. S10†). The identity of H_red_ as resting state in *Tam* HydS is illustrated by the pronounced persistence of H_red_ when H_2_ was removed from the gas phase (*t* > 16.5 min) whereas *Dd* HydAB immediately converted into H_ox_. This accumulation of H_ox_ is a consequence of auto-oxidation, *i.e.* due to H_2_ release. In the next step, the influence of temperature on the H_red_ to H_ox_ transition of *Tam* HydS and *Dd* HydAB was investigated. We addressed the kinetics of auto-oxidation for five temperature points in the range between 20–40 °C. The enzymes were reduced in the presence of 1% H_2_ and subjected to pure N_2_ for 10 min, before they were re-reduced with 1% H_2_. Fig. 6B depicts the changing population of H_ox_ in *Tam* HydS as a function of gas, time, and temperature. Higher temperature increased the rate of H_ox_ formation, upon removal of H_2_ from the atmosphere, and induced a higher percentage of H_ox_ accumulation (*i.e.*, after 10 min). The same set of experiments was performed for *Dd* HydAB. Albeit apparent instability of the H-cluster in *Dd* HydAB at *T* > 30 °C prevented a complete study, the net-oxidation rate in *Dd* HydAB is significantly higher than in *Tam* HydS (Fig. S11†). Moreover, it should be noted that an increase in temperature also resulted in an increase in steady-state concentration of H_ox_ already when *Tam* HydS equilibrated under 1% H_2_, highlighting a positive entropy contribution for the H_red_ to H_ox_ transition (Fig. 6B).

**Fig. 7:**
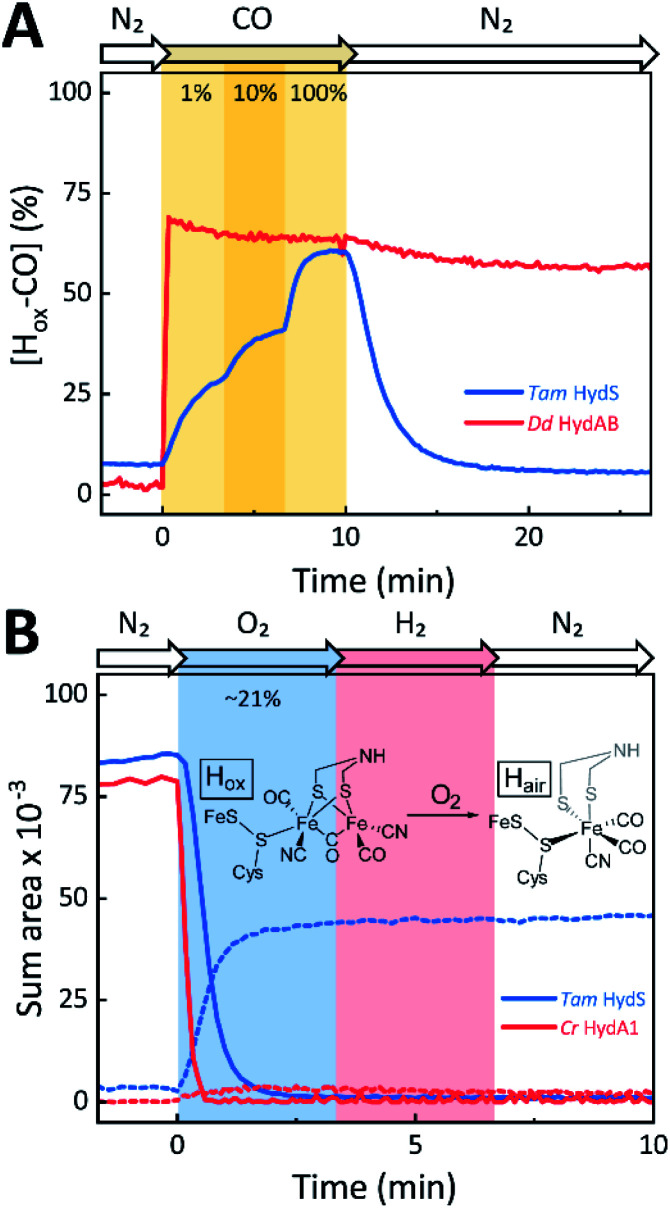
The reactivity of *Tam* HydS and prototypical [FeFe]-hydrogenase (*Dd* HydAB or *Cr* HydA1) towards CO (A) and O_2_ (B) monitored by time-resolved ATR FTIR spectroscopy. (A) Kinetic traces of H_ox_-CO for *Tam* HydS (blue) and *Dd* HydAB (red) that show the reaction with different concentrations of CO. About 30% of the *Dd* HydAB sample were arrested in the CO-insensitive H_inact_ state. Thus, the observed accumulation of ∼70% H_ox_-CO can be considered complete. (B) Kinetic traces for *Tam* HydS (blue) and *Cr* HydA1 (red) that show the reaction with ∼21% O_2_ (air). Solid traces depict H_ox_. While nearly 100% of the H-cluster is lost in *Cr* HydA1, a stable Fe(CO)_2_CN species prevails in *Tam* HydS (dashed traces). The proposed reaction between the oxidized H-cluster (H_ox_) and O_2_ is depicted. FeS = [4Fe–4S]_H_.

The reactivity towards known [FeFe]-hydrogenase inhibitors was probed by exposing protein films to CO or O_2_. Fig. 7A depicts the conversion of H_ox_ into H_ox_-CO for *Tam* HydS and *Dd* HydAB at 1%, 10%, and 100% CO over N_2_. Here, *Tam* HydS displays a notable lack of CO inhibition. Even under 100% CO, only 60% of the H-cluster population converted into H_ox_-CO. Similar trends were observed with 10% H_2_ in the N_2_ carrier gas (Fig. S10†). In the absence of CO gas, H_red_ recovered quickly. Adjusted for the CO-insensitive contamination of ∼30% H_inact_ (Fig. S10†), *Dd* HydAB showed immediate, complete, and enduring CO inhibition. Fig. 7B depicts the reaction of oxidized *Tam* HydS and *Cr* HydA1 with 1 atm air. We chose *Cr* HydA1 for comparison because *Dd* HydAB partly converted into unready states like H_inact_ in the presence of O_2_ whereas O_2_ exposure rapidly destroyed the H-cluster in *Cr* HydA1 (Fig. S12†). In contrast, *Tam* HydS converted into an unprecedented species, denoted H_air_, and the formation of this state was observed regardless of whether the H-cluster resided in the H_red_ or H_ox_ state upon O_2_ exposure (Fig. S9†). As seen in Fig. 5A (blue spectrum) this new state featured two bands in the CO region and one band in the CN^−^ region of the spectrum, suggestive of partial degradation of the [2Fe]_H_ subsite. Moreover, it was found to be unreactive towards N_2_, H_2_, and CO. EPR samples collected of *Tam* HydS exposed to air did not reveal any discernable EPR signal, apart from minor features at *g* ≈ 4.3 and 2.02, attributable to small amounts of Fe^3+^ ions (“junk iron”) and [3Fe–4S] cluster species, respectively. Similarly, a NaDT reduced anaerobic sample of H_air_ was also essentially EPR silent, albeit trace amounts of a [4Fe–4S]^+^ species became discernable (Fig. S13†). A mononuclear version of the [2Fe]_H_ subsite has previously been observed by X-ray crystallography in the prototypical *Cp*I [FeFe]-hydrogenase, following extended O_2_ exposure of the enzyme *in cristallo*.^57^ The overall FTIR spectral features in combination with ^13^CO isotope editing clearly supports the assignment of a mononuclear Fe(CO)_2_CN species (Fig. S14†). Spectro-electrochemistry also suggests that this mononuclear complex is bound to the [4Fe–4S]_H_ cluster and that the modified H-cluster displays at least one redox transition, enabling accumulation of “H_air_-red” and “H_air_-ox” (Fig. 5B, S14 and S15†).

### The catalytic properties of *Tam Hyd*S

**Fig. 8:**
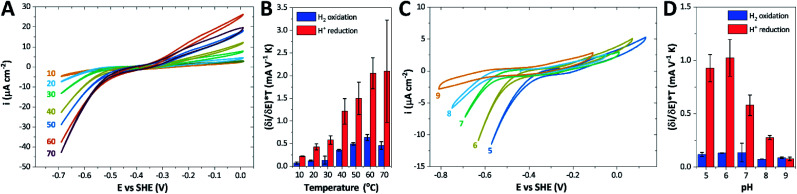
CVs obtained at a rotating disc PGE modified with *Tam* HydS under 1 atm H_2_ at (A) various temperatures in the 10–70 °C range at pH 7 and (C) various pH values from 5 to 9 at 30 °C. The scan rate is 2 mV s^−1^, the rotation rate is 3000 rpm. The data shown in (A) and (C) are obtained from single films cycled up and down in temperature and pH, respectively. Film stability was verified at the end of each experiment by returning the solution to its starting state (pH 7 solution at 30 °C). Dependence of the high driving force slopes of the voltammograms (eqn (1)) times the temperature for H_2_ oxidation and H^+^ reduction on (B) temperature and (D) pH. Error bars show standard deviation between three films.

The catalytic properties of *holo-Tam* HydS were investigated using protein film electrochemistry (PFE), revealing pH and temperature dependent catalytic currents for both H^+^ reduction and H_2_ oxidation (Fig. 8A). Four different procedures were tested for electrode immobilization of the enzyme with the best method being absorption on a pyrolytic graphite electrode in the presence of the polycationic polymyxin B sulfate (Fig. S16†). The observation that the enzyme favors interaction with a positively charged surface suggests that it has a negative net surface charge at the pH of immobilization (pH 7, theoretical pI = 5.87, ExPASy ProtParam tool).

It is important to note that in all experiments even at large over-potentials for both H^+^ reduction and H_2_ oxidation the catalytic current does not reach a steady-state value, but increases almost linearly with over-potential. Such behavior has been rationalized by disorder among the adsorbed enzyme molecules, resulting in a dispersion of interfacial electron transfer rate constants.^58–60^ In this case, the steady-state limiting current (*i*_lim_) can be estimated from a linear fit of the high driving force part of the cyclic voltammograms (CVs), where the slope (∂*i*/∂*E*) is:^60,61^1
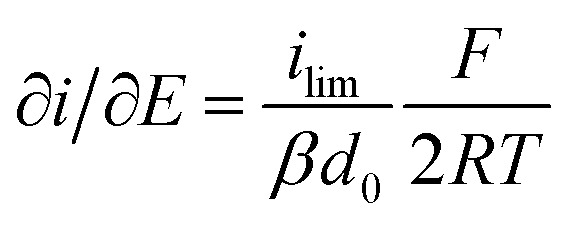


Eqn (1) predicts that the product of the slope and temperature is proportional to the limiting current and therefore to the activity.

We first evaluated the enzyme affinity towards H_2_. It has been noted earlier that 
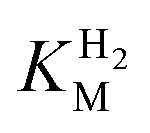
 values determined from PFE experiments can be potential-dependent.^62,63^ Therefore, we recorded CVs at various concentrations of H_2_ in a broad potential window (Fig. S17†). For 
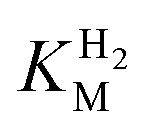
 estimation it is important to measure the current response under conditions where it is limited by the catalytic rate of the enzyme, *i.e.* proportional to the catalytic rate rather than mass transport or interfacial electron transfer.^64^ Thus, 
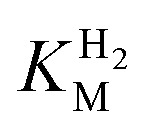
 values at various over-potentials were calculated, and the measurements were performed at 30 and 60 °C. Moreover, to ensure that the catalytic rate is not limited by mass transport, CVs were recorded at rotation rates of 2000 and 3000 rpm at each concentration of H_2_. At over-potentials starting from 200 mV (30 °C) and 100 mV (60 °C), calculated 
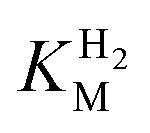
 values were identical within error (Table 2), indicating that the observed current is dominated by the catalytic reaction. Similar 
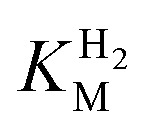
 values were obtained from linear fits of the high driving force part of the cyclic voltammograms at various H_2_ concentrations, further confirming prevalence of the catalytic reaction over interfacial electron transfer at high driving forces (Table S3†).

**Table 2:** 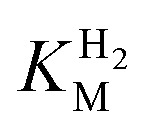
-values for *Tam* HydS determined at various overpotentials and temperatures at pH 7

Over-potential (mV)	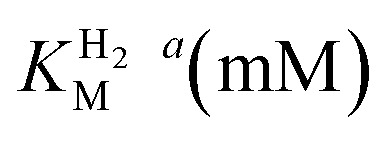	
	30 °C	60 °C
100	0.05 ± 0.01	0.15 ± 0.05
200	0.08 ± 0.02	0.12 ± 0.02
300	0.10 ± 0.02	0.13 ± 0.03
400	0.10 ± 0.03	0.15 ± 0.05
**Average**	**0.09 ± 0.03**	**0.14 ± 0.05**

a The errors for over-potentials 100–400 mV show standard deviation between three films.

We further scrutinized the effect of temperature and pH on the catalytic activity of *holo-Tam* HydS under conditions when the catalytic current for H_2_ oxidation is not limited by mass transport (3000 rpm rotation speed and 1 atm H_2_). Fig. 8A and C displays CVs recorded at various temperatures (10–70 °C) at pH 7, and different pH values at 30 °C, respectively. Fig. 8B shows the temperature dependence of the CVs at high driving force (eqn (1)) for H^+^ reduction and H_2_ oxidation. Fig. 8D shows the corresponding data as a function of pH. When pH is decreased, the catalytic activity towards H^+^ reduction increases and the catalytic wave is shifted to more anodic potentials, consistent with higher H^+^ concentration (Fig. 8C). Conversely, the magnitude of the catalytic current for H_2_ oxidation does not vary smoothly with pH. The oxidation process is pH independent between pHs 8–9, increases at pH 6–7 and remains stable at this higher current down to pH 5. Moreover, over-potential for both H_2_ oxidation and H^+^ reduction is lowest at pH 5 (Fig. S18†). The origin of this pH switching behavior for H_2_ oxidation is currently not identified.

**Fig. 9:**
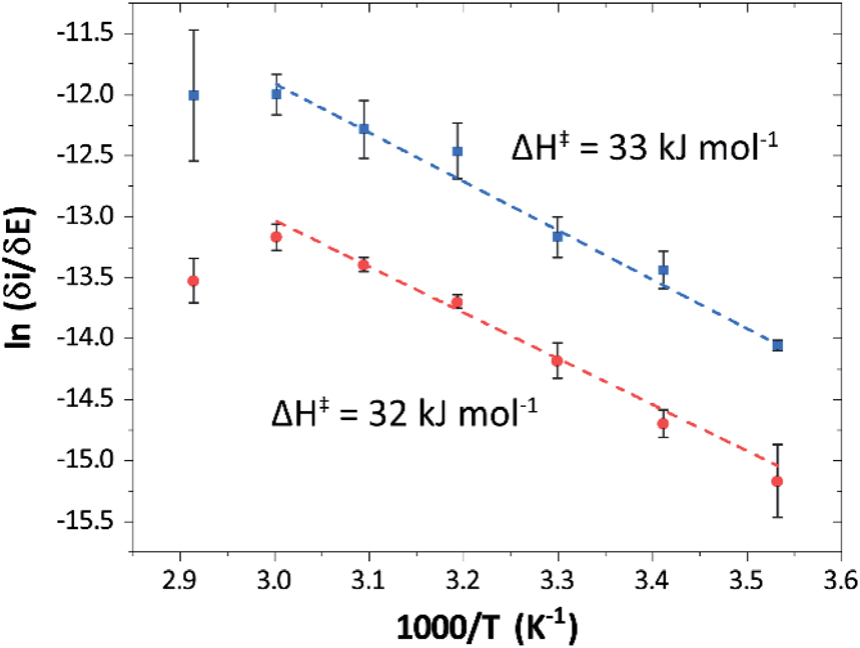
Eyring plots for H^+^ reduction (red) and H_2_ oxidation (blue) with linear fits (dashed lines). Plots prepared using estimated *i*_lim_ based on data from Fig. 8B (data observed at 70 °C excluded from the linear fit).

Temperature was found to have a strong influence on H_2_ oxidation and H^+^ reduction between 10–60 °C at pH 7 (Fig. 8A). Increasing the temperature not only resulted in higher overall currents, but also a significant decrease in over-potential. At room temperature, an over-potential of 50–60 mVs was observed in both catalytic directions, in contrast to previously characterized group A [FeFe]-hydrogenases. With the exception of specific mutants the latter enzymes generally display rapid increase in currents around the thermodynamic midpoint potential.^44,65,66^ The over-potential requirement of *Tam* HydS is in line with the quasi-reversible nature of the H_ox_/H_red_ transition observed by FTIR spectro-electrochemistry (Fig. S8†). At temperatures of 50–60 °C the over-potential decreased to approx. 10 mV. The decrease of catalytic currents at 70 °C is attributed to the deactivation of the protein, since we did not observe any significant protein loss during the experiment at temperatures up to 60 °C (Fig. S19†). It should also be noted that no oxidative inactivation^67,68^ was observed when cycling up to ±0 mV *vs.* SHE. Stability and increased catalytic activity of the protein at elevated temperatures is not surprising considering the thermophilic nature of *T. mathranii*.^69^ The activation enthalpies (Δ*H*^‡^) of the H_2_ oxidation and H^+^ reduction reactions were estimated through Eyring plots based on the change of the high potential slope as a function of temperature (Fig. 9), and found to be similar in both catalytic directions (Table 3). In the case of the prototypical [FeFe]-hydrogenases *Cr* HydA1 and *Ca*I (Table 3),^70^ distinctly lower activation enthalpies (Δ*H*^‡^) for either H^+^ reduction or H_2_ oxidation, respectively, have been reported, suggesting that *Tam* HydS is exceptionally well balanced for bidirectional catalysis. Moreover, albeit the activation enthalpies are higher for *Tam* HydS than *Cr* HydA1 and *Ca*I, they are still significantly lower than what has been reported from the *E. coli* [NiFe]-hydrogenases *Ec* Hyd1 and *Ec* Hyd2 (Table 3). Thus, the low specific activity observed for *Tam* HydS (Fig. 2) cannot be explained by differences in activation enthalpies alone. Rather, the low catalytic rate of *Tam* HydS is governed by mass transfer, *e.g.* proton- or H_2_ transfer within the protein. Impaired proton transfer could also, at least partially, explain the over-potential observed at low temperature.^44^ Finally, it is noteworthy that films prepared of the enzyme exposed to air, to induce the formation of the H_air_ state, displayed limited capacity for H^+^ reduction but a complete loss of H_2_ oxidation function (Fig. S20†). The catalytic properties of H_air_ was further supported by *in vitro* assays, showing a H^+^ reduction activity approximately hundred-fold lower than the native enzyme.

**Table 3:** Activation enthalpies (Δ*H*^‡^) for H^+^ reduction and H_2_ oxidation observed for different [FeFe]- and [NiFe]-hydrogenases

Enzyme	Δ*H*^‡^ (kJ mol^−1^)	
	H^+^ reduction	H_2_ oxidation
*Tam* HydS (this work)	32 ± 3	33 ± 2
*Ca*I^a^	29	19
*Cr* HydA1^a^	20^b^	26^b^
*Ec* Hyd1^a^^,^^c^	—	48
*Ec* Hyd2^a^^,^^d^	65	37

a Experimental data obtained from ref. 62, determined at pH 6, 30 °C.

b Only reached at high over-potential.

c [NiFe]-hydrogenase *E. coli* Hyd1.

d [NiFe]-hydrogenase *E. coli* Hyd2.

## Conclusions

This report represents the first biochemical and biophysical characterization of a group D [FeFe]-hydrogenase. As described herein, *Tam* HydS features a number of properties similar to regulatory [NiFe]-hydrogenases,^71^ including a relatively low 
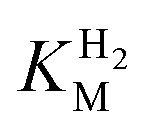
 and an increased tolerance against CO inhibition. This is in line with a potential sensory function, as expected from its close relationship to the putatively sensory group C [FeFe]-hydrogenases. Still, a 
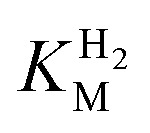
 of around 0.1 mM is 5–10 times higher than what has been reported for regulatory [NiFe]-hydrogenases,^71,72^ but is arguably in agreement with the overall higher 
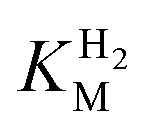
 and 
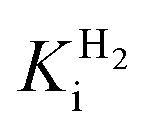
 reported for prototypical [FeFe]-hydrogenase.^73^ Moreover, analysis on a genomic level shows that *Tam* HydS is encoded upstream of a heterotrimeric bifurcating group A [FeFe]-hydrogenase. Thus, we hypothesize that M2e represents an alternative type of sensory [FeFe]-hydrogenase. This raises the question of how signal transduction is achieved. In the case of group C putative sensory [FeFe]-hydrogenase, a PAS-domain is fused to the H-domain. Similarly, to the best of our knowledge, all regulatory [NiFe]-hydrogenases identified to-date feature the so-called HoxJ subunit, encoding a PAS domain-like sequence. A PAS-domain cannot be identified in the operon encoding for *Tam* HydS but the gene is flanked by a histidine kinase-like ATPase and an AraC family transcriptional regulator. These genes are commonly involved in signal transduction and regulation of transcription, which further suggests a regulatory role for the M2e hydrogenases.^74,75^

In the context of H-cluster function, *Tam* HydS displays a number of diverging properties. As reflected in the tolerance towards CO, the unusual mixture of H_ox_-like states, reaction enthalpies (Δ*H*^‡^) that were practically identical for both H^+^ reduction and H_2_ oxidation, and a stabilization of the H_red_-state. Moreover, it is noteworthy that despite its high tolerance towards CO *Tam* HydS reacted rapidly with O_2_ to form a stable mononuclear version of the [2Fe]_H_ subsite (H_air_). This argues against gas diffusion (mass transfer) to the active site as the main defense mechanism towards CO inhibition, which we instead attribute to the unusual hydrogen-bonding network around the H-cluster afforded by variations of *e.g.* Cys299, Met353 and Met497 (Fig. 1). Indeed, an increased resistance towards CO has also been reported for the putative-sensory group C [FeFe]-hydrogenase *Tm* HydS, featuring different alterations of the same residues.^38^ The influence of the hydrogen-bonding network on CO affinity has also been suggested by studies of cofactor variants and mutants of *Cr* HydA1, in which the nearby cysteine was replaced by an alanine residue.^51^

The effect of O_2_ on prototypical group A [FeFe]-hydrogenases has been intensively studied, nevertheless formation of H_air_ has not been reported for any other [FeFe]-hydrogenase. Instead, the reaction generally causes complete H-cluster degradation or formation of reversibly inhibited states like H_inact_ by coordination of thiol ligands to the [2Fe] subsite.^37,76^ Whether H_air_ has a physiological role remains uncertain. However, as this state appears unreactive towards H_2_ while retaining a limited H_2_ production capacity, it is unlikely to be relevant in H_2_ sensing. Still, its formation provides a striking example of how the active site environment modulates the reactivity of the H-cluster. It should be noted that formation of H_air_ is dependent on O_2_ as the equivalent state does not seem to be formed under oxidizing electrochemical conditions. As O_2_ acts as a chemical oxidant it can lead to an alternative pathway that remains to be fully elucidated. Arguably, access to electrons and protons has a large influence on the reaction between the H-cluster and O_2_ and several factors need to be considered to explain the formation of H_air_ in *Tam* HydS.^77,78^ It has been shown that in the case of [NiFe]-hydrogenase, O_2_ can be efficiently reduced to H_2_O through rapid electron injection to the [NiFe] cofactor involving an unusual [4Fe–4S] cluster.^79^ The presence of a C-terminal [4Fe–4S] cluster and a disrupted proton transfer pathway differentiates M2e enzymes from prototypical [FeFe]-hydrogenases. Still, formation of H_air_ was not reported in the case of *Tm* HydS which features the same differences.^38^ This suggests that the reaction between the H-cluster and O_2_ is significantly influenced by other variations in the active site architecture.

We presume that the lack of a cysteine in position 299 also influences the catalytic activity of *Tam* HydS, as hindered proton release could stabilize H_red_ kinetically, following reduction of the H-cluster with H_2_. A similar stabilization of H_red_ has been noted for *Tm* HydS.^38^ However, while the latter enzyme is suggested to form an unusual, unprotonated, CO-bridged form of H_red_
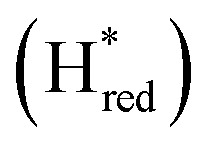
, *Tam* HydS appears to form an H_red_ state highly similar to that of prototypical [FeFe]-hydrogenase. Also, the complete lack of the well-conserved proton transfer pathway found in group A [FeFe]-hydrogenases raises the question of where an alternative pathway could reside. In the homology model of *Tam* HydS, a conserved glutamate residue close to the H-cluster (Glu252) is located in a preferable position for initiating proton transfer (Fig. S21†). However, there is no clear continuation of this potential pathway. In addition to changes in the proton transfer pathway, variation of Met353 and Met497 certainly also modulates the electronic properties of the H-cluster. It has been previously proposed that Met353 is important for tuning 
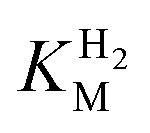
 as well as the catalytic bias of prototypical [FeFe]-hydrogenase, in favor of either H^+^ reduction or H_2_ oxidation.^31,41^ Still, the exact contributions of the Met353Ser variation on the activity of *Tam* HydS remain to be fully elucidated.

In closing, this study shows that the *Tam* HydS M2e enzyme displays significant differences in reactivity as compared to previously studied group A as well as group C [FeFe]-hydrogenases. Thus, in addition to proposing a biological function for the group D [FeFe]-hydrogenases, it underscores how mutations of known [FeFe]-hydrogenases need to be complemented with further studies of the biodiversity to fully realize the chemical space of this fascinating family of enzymes. Mapping out the reactivity of these diverse enzymes is certainly critical for our understanding of hydrogen metabolism and envisioned biotechnological applications. It is also of high relevance in the context of bioinspired catalyst design, as it will provide new model systems for elucidating the influence of the protein environment and the outer coordination sphere on the reactivity of the H-cluster.

## Conflicts of interest

There are no conflicts to declare.

## Supplementary Material

SC-011-D0SC03319G-s001

## References

[cit1] Madden C., Vaughn M. D., Díez-Pérez I., Brown K. A., King P. W., Gust D., Moore A. L., Moore T. A. (2012). J. Am. Chem. Soc..

[cit2] Hatchikian E. C., Forget N., Fernandez V. M., Williams R., Cammack R. (1992). Eur. J. Biochem..

[cit3] Tard C., Pickett C. J. (2009). Chem. Rev..

[cit4] Simmons T. R., Berggren G., Bacchi M., Fontecave M., Artero V. (2014). Coord. Chem. Rev..

[cit5] Calusinska M., Happe T., Joris B., Wilmotte A. (2010). Microbiology.

[cit6] Meyer J. (2007). Cell. Mol. Life Sci..

[cit7] Greening C., Biswas A., Carere C. R., Jackson C. J., Taylor M. C., Stott M. B., Cook G. M., Morales S. E. (2016). ISME J..

[cit8] Benoit S. L., Maier R. J., Sawers R. G., Greening C. (2020). Microbiol. Mol. Biol. Rev..

[cit9] Land H., Senger M., Berggren G., Stripp S. T. (2020). ACS Catal..

[cit10] Peters J. W., Lanzilotta W. N., Lemon B. J., Seefeldt L. C. (1998). Science.

[cit11] Nicolet Y., Piras C., Legrand P., Hatchikian C. E., Fontecilla-Camps J. C. (1999). Structure.

[cit12] Silakov A., Wenk B., Reijerse E., Lubitz W. (2009). Phys. Chem. Chem. Phys..

[cit13] Berggren G., Adamska A., Lambertz C., Simmons T. R., Esselborn J., Atta M., Gambarelli S., Mouesca J. M., Reijerse E., Lubitz W., Happe T., Artero V., Fontecave M. (2013). Nature.

[cit14] Kamp C., Silakov A., Winkler M., Reijerse E. J., Lubitz W., Happe T. (2008). Biochim. Biophys. Acta, Bioenerg..

[cit15] Happe T., Naber J. D. (1993). Eur. J. Biochem..

[cit16] Albracht S. P. J., Roseboom W., Hatchikian E. C. (2006). J. Biol. Inorg Chem..

[cit17] Roseboom W., De Lacey A. L., Fernandez V. M., Hatchikian E. C., Albracht S. P. J. (2006). J. Biol. Inorg Chem..

[cit18] Bennett B., Lemon B. J., Peters J. W. (2000). Biochemistry.

[cit19] Verhagen M. F., O'Rourke T., Adams M. W. (1999). Biochim. Biophys. Acta, Bioenerg..

[cit20] Chongdar N., Pawlak K., Rüdiger O., Reijerse E. J., Rodríguez-Maciá P., Lubitz W., Birrell J. A., Ogata H. (2020). J. Biol. Inorg Chem..

[cit21] Mulder D. W., Shepard E. M., Meuser J. E., Joshi N., King P. W., Posewitz M. C., Broderick J. B., Peters J. W. (2011). Structure.

[cit22] Lubitz W., Ogata H., Rüdiger O., Reijerse E. (2014). Chem. Rev..

[cit23] Birrell J. A., Pelmenschikov V., Mishra N., Wang H., Yoda Y., Tamasaku K., Rauchfuss T. B., Cramer S. P., Lubitz W., DeBeer S. (2020). J. Am. Chem. Soc..

[cit24] Haumann M., Stripp S. T. (2018). Acc. Chem. Res..

[cit25] Sommer C., Adamska-Venkatesh A., Pawlak K., Birrell J. A., Rüdiger O., Reijerse E. J., Lubitz W. (2017). J. Am. Chem. Soc..

[cit26] Mulder D. W., Guo Y., Ratzloff M. W., King P. W. (2017). J. Am. Chem. Soc..

[cit27] Mulder D. W., Ratzloff M. W., Bruschi M., Greco C., Koonce E., Peters J. W., King P. W. (2014). J. Am. Chem. Soc..

[cit28] Reijerse E. J., Pham C. C., Pelmenschikov V., Gilbert-Wilson R., Adamska-Venkatesh A., Siebel J. F., Gee L. B., Yoda Y., Tamasaku K., Lubitz W., Rauchfuss T. B., Cramer S. P. (2017). J. Am. Chem. Soc..

[cit29] Mészáros L. S., Ceccaldi P., Lorenzi M., Redman H. J., Pfitzner E., Heberle J., Senger M., Stripp S. T., Berggren G. (2020). Chem. Sci..

[cit30] Adamska-Venkatesh A., Krawietz D., Siebel J., Weber K., Happe T., Reijerse E., Lubitz W. (2014). J. Am. Chem. Soc..

[cit31] Artz J. H., Zadvornyy O. A., Mulder D. W., Keable S. M., Cohen A. E., Ratzloff M. W., Williams S. G., Ginovska B., Kumar N., Song J., McPhillips S. E., Davidson C. M., Lyubimov A. Y., Pence N., Schut G. J., Jones A. K., Soltis S. M., Adams M. W. W., Raugei S., King P. W., Peters J. W. (2020). J. Am. Chem. Soc..

[cit32] Caserta G., Papini C., Adamska-Venkatesh A., Pecqueur L., Sommer C., Reijerse E., Lubitz W., Gauquelin C., Meynial-Salles I., Pramanik D., Artero V., Atta M., del Barrio M., Faivre B., Fourmond V., Léger C., Fontecave M. (2018). J. Am. Chem. Soc..

[cit33] Gauquelin C., Baffert C., Richaud P., Kamionka E., Etienne E., Guieysse D., Girbal L., Fourmond V., Andre I., Guigliarelli B., Leger C., Soucaille P., Meynial-Salles I. (2018). Biochim. Biophys. Acta, Bioenerg..

[cit34] Butt J. N., Filipiak M., Hagen W. R. (1997). Eur. J. Biochem..

[cit35] Morra S., Arizzi M., Valetti F., Gilardi G. (2016). Biochemistry.

[cit36] Baffert C., Demuez M., Cournac L., Burlat B., Guigliarelli B., Bertrand P., Girbal L., Léger C. (2008). Angew. Chem., Int. Ed..

[cit37] Rodríguez-Maciá P., Reijerse E. J., van Gastel M., DeBeer S., Lubitz W., Rüdiger O., Birrell J. A. (2018). J. Am. Chem. Soc..

[cit38] Chongdar N., Birrell J. A., Pawlak K., Sommer C., Reijerse E. J., Rüdiger O., Lubitz W., Ogata H. (2018). J. Am. Chem. Soc..

[cit39] Rodríguez-Maciá P., Pawlak K., Rüdiger O., Reijerse E. J., Lubitz W., Birrell J. A. (2017). J. Am. Chem. Soc..

[cit40] Land H., Ceccaldi P., Mészáros L. S., Lorenzi M., Redman H. J., Senger M., Stripp S. T., Berggren G. (2019). Chem. Sci..

[cit41] Knörzer P., Silakov A., Foster C. E., Armstrong F. A., Lubitz W., Happe T. (2012). J. Biol. Chem..

[cit42] Duan J., Senger M., Esselborn J., Engelbrecht V., Wittkamp F., Apfel U.-P., Hofmann E., Stripp S. T., Happe T., Winkler M. (2018). Nat. Commun..

[cit43] Senger M., Eichmann V., Laun K., Duan J., Wittkamp F., Knör G., Apfel U.-P., Happe T., Winkler M., Heberle J., Stripp S. T. (2019). J. Am. Chem. Soc..

[cit44] Lampret O., Duan J., Hofmann E., Winkler M., Armstrong F. A., Happe T. (2020). Proc. Natl. Acad. Sci. U. S. A..

[cit45] Pandelia M.-E., Nitschke W., Infossi P., Giudici-Orticoni M.-T., Bill E., Lubitz W. (2011). Proc. Natl. Acad. Sci. U. S. A..

[cit46] Rousset M., Montet Y., Guigliarelli B., Forget N., Asso M., Bertrand P., Fontecilla-Camps J. C., Hatchikian E. C. (1998). Proc. Natl. Acad. Sci. U. S. A..

[cit47] Caserta G., Adamska-Venkatesh A., Pecqueur L., Atta M., Artero V., Roy S., Reijerse E., Lubitz W., Fontecave M. (2016). Biochim. Biophys. Acta, Bioenerg..

[cit48] Esselborn J., Lambertz C., Adamska-Venkatesh A., Simmons T., Berggren G., Noth J., Siebel J., Hemschemeier A., Artero V., Reijerse E., Fontecave M., Lubitz W., Happe T. (2013). Nat. Chem. Biol..

[cit49] Németh B., Esmieu C., Redman H. J., Berggren G. (2019). Dalton Trans..

[cit50] Adamska-Venkatesh A., Simmons T. R., Siebel J. F., Artero V., Fontecave M., Reijerse E., Lubitz W. (2015). Phys. Chem. Chem. Phys..

[cit51] Duan J., Mebs S., Laun K., Wittkamp F., Heberle J., Happe T., Hofmann E., Apfel U.-P., Winkler M., Senger M., Haumann M., Stripp S. T. (2019). ACS Catal..

[cit52] Senger M., Mebs S., Duan J., Shulenina O., Laun K., Kertess L., Wittkamp F., Apfel U.-P., Happe T., Winkler M., Haumann M., Stripp S. T. (2018). Phys. Chem. Chem. Phys..

[cit53] Roseboom W., De Lacey A. L., Fernandez V. M., Hatchikian E. C., Albracht S. P. J. (2006). J. Biol. Inorg Chem..

[cit54] Sommer C., Adamska-Venkatesh A., Pawlak K., Birrell J. A., Rüdiger O., Reijerse E. J., Lubitz W. (2017). J. Am. Chem. Soc..

[cit55] Ratzloff M. W., Artz J. H., Mulder D. W., Collins R. T., Furtak T. E., King P. W. (2018). J. Am. Chem. Soc..

[cit56] Winkler M., Senger M., Duan J., Esselborn J., Wittkamp F., Hofmann E., Apfel U.-P., Stripp S. T., Happe T. (2017). Nat. Commun..

[cit57] Esselborn J., Kertess L., Apfel U.-P., Hofmann E., Happe T. (2019). J. Am. Chem. Soc..

[cit58] Adamska A., Silakov A., Lambertz C., Rüdiger O., Happe T., Reijerse E., Lubitz W. (2012). Angew. Chem., Int. Ed..

[cit59] Jones A. K., Sillery E., Albracht S. P. J., Armstrong F. A. (2002). Chem. Commun..

[cit60] Léger C., Jones A. K., Albracht S. P. J., Armstrong F. A. (2002). J. Phys. Chem. B.

[cit61] Where ilim is the limiting current, *β* is a decay constant, *d*_0_ is a range of the tunneling distances between the electrode and the entry point for electrons in the enzyme, *F* is the Faraday constant, *R* is the gas constant, and *T* is temperature

[cit62] Léger C., Dementin S., Bertrand P., Rousset M., Guigliarelli B. (2004). J. Am. Chem. Soc..

[cit63] Goldet G., Wait A. F., Cracknell J. A., Vincent K. A., Ludwig M., Lenz O., Friedrich B., Armstrong F. A. (2008). J. Am. Chem. Soc..

[cit64] Léger C., Elliott S. J., Hoke K. R., Jeuken L. J. C., Jones A. K., Armstrong F. A. (2003). Biochemistry.

[cit65] Lampret O., Adamska-Venkatesh A., Konegger H., Wittkamp F., Apfel U.-P., Reijerse E. J., Lubitz W., Rüdiger O., Happe T., Winkler M. (2017). J. Am. Chem. Soc..

[cit66] Pandey K., Islam S. T. A., Happe T., Armstrong F. A. (2017). Proc. Natl. Acad. Sci. U. S. A..

[cit67] Vincent K. A., Parkin A., Lenz O., Albracht S. P. J., Fontecilla-Camps J. C., Cammack R., Friedrich B., Armstrong F. A. (2005). J. Am. Chem. Soc..

[cit68] Fourmond V., Greco C., Sybirna K., Baffert C., Wang P.-H., Ezanno P., Montefiori M., Bruschi M., Meynial-Salles I., Soucaille P., Blumberger J., Bottin H., De Gioia L., Léger C. (2014). Nat. Chem..

[cit69] Jayasinghearachchi H. S., Sarma P. M., Lal B. (2012). Int. J. Hydrogen Energy.

[cit70] Hexter S. V., Grey F., Happe T., Climent V., Armstrong F. A. (2012). Proc. Natl. Acad. Sci. U. S. A..

[cit71] Bernhard M., Buhrke T., Bleijlevens B., De Lacey A. L., Fernandez V. M., Albracht S. P. J., Friedrich B. (2001). J. Biol. Chem..

[cit72] Ash P. A., Liu J., Coutard N., Heidary N., Horch M., Gudim I., Simler T., Zebger I., Lenz O., Vincent K. A. (2015). J. Phys. Chem. B.

[cit73] Fourmond V., Baffert C., Sybirna K., Dementin S., Abou-Hamdan A., Meynial-Salles I., Soucaille P., Bottin H., Léger C. (2013). Chem. Commun..

[cit74] Wolanin P. M., Thomason P. A., Stock J. B. (2002). Genome Biol..

[cit75] Gallegos M. T., Schleif R., Bairoch A., Hofmann K., Ramos J. L. (1997). Microbiol. Mol. Biol. Rev..

[cit76] Swanson K. D., Ratzloff M. W., Mulder D. W., Artz J. H., Ghose S., Hoffman A., White S., Zadvornyy O. A., Broderick J. B., Bothner B., King P. W., Peters J. W. (2015). J. Am. Chem. Soc..

[cit77] Kubas A., Orain C., De Sancho D., Saujet L., Sensi M., Gauquelin C., Meynial-Salles I., Soucaille P., Bottin H., Baffert C., Fourmond V., Best R. B., Blumberger J., Léger C. (2017). Nat. Chem..

[cit78] Mebs S., Kositzki R., Duan J., Kertess L., Senger M., Wittkamp F., Apfel U.-P., Happe T., Stripp S. T., Winkler M., Haumann M. (2018). Biochim. Biophys. Acta, Bioenerg..

[cit79] Shafaat H. S., Rüdiger O., Ogata H., Lubitz W. (2013). Biochim. Biophys. Acta, Bioenerg..

[cit80] Sievers F., Wilm A., Dineen D., Gibson T. J., Karplus K., Li W., Lopez R., McWilliam H., Remmert M., Söding J., Thompson J. D., Higgins D. G. (2011). Mol. Syst. Biol..

