# Supplementary material for: Characterization of a putative sensory [FeFe]-hydrogenase provides new insight into the role of the active site architecture
Source: Chem Sci. 2020 Sep 21;11(47):12789–801. doi: 10.1039/d0sc03319g (PMC8163306; doi:10.1039/d0sc03319g)
Supplement: SC-011-D0SC03319G-s001 [file SC-011-D0SC03319G-s001.pdf]

Electronic Supplementary Material (ESI) for Chemical Science.
This journal is © The Royal Society of Chemistry 2020

Supplementary Information

Characterization of a putative sensory [FeFe]-hydrogenase provides new insight into the role of the active site architecture

Henrik Land,[a] Alina Sekretareva,[a] Ping Huang,[a] Holly J. Redman,[a] Brigitta Németh,[a, ‡] Nakia Polidori,[a, ‡‡] Lívia S. Mészáros,[a] Moritz Senger,[b,c] Sven Stripp*[c] and Gustav Berggren*[a]

[a] Molecular Biomimetics, Department of Chemistry – Ångström Laboratory, Uppsala University, Box 523, SE-75120, Uppsala, Sweden

[b] Physical Chemistry, Department of Chemistry – Ångström Laboratory, Uppsala University, Box 523, SE-75120, Uppsala, Sweden

[c] Institute of Experimental Physics, Experimental Molecular Biophysics, Freie Universität Berlin, Arnimallee 14, DE-14195, Berlin, Germany

[‡] Current address: Department of Chemistry and Biochemistry, Montana State University, Bozeman, Montana 59717, USA

[‡‡] Current address: Institute of Molecular Biosciences, University of Graz, Humboldtstrasse 50, 8010 Graz, Austria

E-mail: Gustav.Berggren@kemi.uu.se

Table of Contents

Experimental procedures 2
General 2
Protein expression and purification 2
Aerobic purification 2
Anaerobic purification 2
Iron-sulfur cluster reconstitution 2
H_2-production assay 3
Homology modeling 3
EPR sample preparation 3
EPR spectroscopy 3
Protein film electrochemistry sample preparation 3
Protein film electrochemistry 3
FTIR sample preparation 4
FTIR spectroscopy 4
Supplementary tables and figures 5
Figure S1. SDS-PAGE of purified Tam HydS 5
Figure S2. Schematic overview of the ATR-FTIR setup 5
Figure S3. g-tensor assignment of [4Fe4S] clusters. 6
Figure S4. Power saturation study of the EPR signals of *holo-Tam* HydS. 7
Figure S5. Supporting EPR data on *holo-Tam* HydS (effect of pH and reductant; and simulations) 8
Figure S6. FTIR spectroscopic characterization of pdt-*Tam* HydS 9
Figure S7. Accumulation of $H_{ox}H$ at low pH in the presence of reductant. 10
Figure S8. ATR-FTIR spectro-electrochemical study of the H_{ox}/H_{red} interconversion. 11
Figure S9. Formation of H_{air} and H_{ox}-CO under oxidizing and reducing conditions. 12
Figure S10. Full kinetic traces for the reaction with H_2 and/or CO in *Tam* HydS and *Dd* HydAB. 13
Figure S11. Full kinetic traces for the evaluation of the temperature effect in *Tam* HydS and *Dd* HydAB. 14
Figure S12. Accumulation of super-oxidized states in *Dd* HydAB under O_2. 15
Table S1. IR signature of H-cluster states observed in *Dd* HydAB (CO-only) 15
Figure S13. EPR-spectra in the g ≈ 2 region of as prepared and reduced H_{air} 16
Figure S14. ^{13}CO isotope editing and spectroelectrochemical characterization of H_{air}-ox and H_{air}-red. 17
Table S2. Isotopomers generated in this study. 17
Figure S15. Suggested geometry of H_{air}. 18
Figure S16. Optimization of the immobilization protocol in PFE. 19
Figure S17. Influence of H_2 partial pressure on currents observed in PFE. 20
Figure S18. Influence of pH on currents observed in PFE. 21
Figure S19. Stability of protein film in PFE. 22
Figure S20. Reduction currents observed for H_{air} 23
Table S3. $K_M^{H_2}$ calculated using estimated i_{lim}. 23
Figure S21. Active site of *Tam* HydS homology model 24
References 25

Experimental procedures

General

All chemicals were purchased from Sigma-Aldrich or VWR and used as received unless otherwise stated. Protein expression was analyzed by 12% SDS-PAGE minigels in a BIO-RAD Mini PROTEAN® system. The proteins were stained with Page Blue protein staining solution (Thermo Fisher Scientific) according to the supplier instructions. All anaerobic work was performed in an MBRAUN glovebox ([O_2] < 10 ppm). The $[2Fe]^{adt}$ and $[2Fe]^{pdt}$ subsite mimics were synthesized in accordance to literature protocols with minor modifications, and verified by FTIR spectroscopy.[1-5]

Protein expression and purification

The gene encoding for *Tam* HydS was synthesized and cloned in pET-11a(+) by Genscript® using restriction sites NdeI and BamHI following codon optimization for expression in *E. coli*. Transformation of the plasmid into *E. coli* was performed using heat shock at 42°C. Transformed *E. coli* were grown overnight in 10 mL LB medium containing 100 µg mL^{-1} ampicillin at 37°C. These cultures were subsequently used to inoculate 1 L of M9 medium (22 mM Na_2HPO_4, 22 mM KH_2PO_4, 85 mM NaCl, 18 mM NH_4Cl, 0.2 mM $MgSO_4$, 0.1 mM $CaCl_2$, 0.4% (v/v) glucose) containing 100 µg mL^{-1} ampicillin. Cultures were grown at 37°C and 150 rpm until an optical density (OD_{600}) of appr. 0.4-0.6 was reached. Protein expression was induced by addition of 0.1 mM $FeSO_4$ and 1 mM isopropyl β-D-1-thiogalactopyranoside (IPTG) and cultures were incubated at 20°C and 150 rpm for appr. 16 h. Cells were thereafter harvested by centrifugation in a Beckman Coulter Avanti J-25 centrifuge (5,000 rpm/4,424 x g, 10 min). *Cr HydA1* was prepared as previously described.[6] *Dd* HydAB was kindly provided by Prof. Juan Fontecilla-Camps.

Aerobic purification

The cell pellet was resuspended in lysis buffer (Tris-HCl (100 mM, pH 8), NaCl (150 mM), $MgCl_2$ (10 mM), lysozyme from chicken egg white (1 mg mL^{-1}), DNAse I from bovine pancreas (0.05 mg mL^{-1}) and RNAse A from bovine pancreas (0.05 mg mL^{-1}) and incubated on ice for 30 min. Cell lysis was performed by three cycles of freezing/thawing in liquid N_2 followed by sonication. Cell debris was removed by centrifugation in a Beckman Coulter Optima L-90K Ultracentrifuge (55,000 rpm/222,592 x g, 60 min) and the supernatant was collected and filtered (0.45 µm syringe filter) before being loaded on a StrepTrap™ HP (GE Healthcare) affinity column using an ÄKTA pure FPLC system (GE Healthcare) and purified according to the manufacturer´s instructions.

Anaerobic purification

The cell pellet was brought into an anaerobic glovebox, resuspended in lysis buffer (Tris-HCl (100 mM, pH 8), NaCl (150 mM), $MgCl_2$ (10 mM), lysozyme from chicken egg white (1 mg mL^{-1}), DNAse I from bovine pancreas (0.05 mg mL^{-1}) and RNAse A from bovine pancreas (0.05 mg mL^{-1}) and incubated on ice for 30 min. Cell lysis was performed by three cycles of freezing/thawing in liquid N_2. Cell debris was removed by centrifugation in a Beckman Coulter Optima L-90K Ultracentrifuge (55,000 rpm/222,592 x g, 60 min) and the supernatant was collected and filtered (0.45 µm syringe filter) before being loaded on a StrepTrap™ HP (GE Healthcare) affinity column using a BioLogic DuoFlow™ FPLC system (Bio-Rad) and purified according to the manufacturer´s instructions.

The concentration of all purified proteins was quantified using the Bradford assay with bovine serum albumin as a protein standard.[7] Quantification of Fe-content was performed using a previously reported assay.[8]

Iron-sulfur cluster reconstitution

Iron-sulfur cluster reconstitution was performed under strictly anaerobic conditions and was preceded by a 10-minute incubation with a tenfold molar excess of dithiothreitol at room temperature. Ferrous ammonium sulfate and L-cysteine was then added in a 1.5-fold molar excess to the desired amount of Fe-atoms to be added (dependent on the initial Fe-content in the purified protein). Reconstitution was initiated by the addition of 1% molar equivalent of cysteine desulfarase (CsdA) from *E. coli*. The reaction was followed by monitoring the absorbance increase around 400 nm by UV/Visible spectroscopy using an AvaSpec-ULS2048-USB2-UA-50: Avantes Fiber Optic UV/VIS/NIR spectrometer. After the reconstitution was finished, it was stopped by running the reaction through a PD-10 column (GE Healthcare).

H_2-production assay

Activation of *Tam* HydS with subsequent measurement of H_2-production activity was performed under strictly anaerobic conditions by mixing *apo-Tam* HydS (1 µM) with sodium dithionite (20 µM, 20x excess), $[2Fe]^{adt}$ (12 µM, 12x excess) and Triton X-100 (1% v/v) in sodium phosphate buffer (100 mM, pH 6.8). The activation mixture was incubated at room temperature for 1 h. The reaction was then initiated by the rapid addition of methyl viologen (10 mM) and sodium dithionite (100 mM). The reaction was incubated at 30°C for 15 min. The amount of produced H_2 was then determined by analyzing the reaction headspace on a PerkinElmer Clarus 500 gas chromatograph (GC) equipped with a thermal conductivity detector (TCD) and a stainless-steel column packed with Molecular Sieve (60/80 mesh). The operational temperatures of the injection port, the oven and the detector were 100 °C, 80 °C and 100 °C, respectively. Argon was used as carrier gas at a flow rate of 35 mL min^{-1}.

Homology modeling

Homology modeling of truncated *Tam* HydS was performed using YASARA Structure version 18.3.23 as previously described.[9]

EPR sample preparation

EPR samples were prepared under strictly anaerobic conditions by diluting reconstituted *Tam* HydS to 50 µM in Tris-HCl buffer (100 mM, pH 8). Activated samples were treated with 600 µM $[2Fe]^{adt}$ or $[2Fe]^{pdt}$ (12x excess) and reduced samples were treated with 1 mM sodium dithionite (20x excess). Samples were then incubated at room temperature for 30 min. All gas treated samples were exposed to the specific gas (N_2, H_2 or air) for 10 min. Samples were finally transferred to EPR-tubes and flash-frozen in liquid N_2.

EPR spectroscopy

X-band EPR measurements were performed on a Bruker ELEXYS E500 spectrometer equipped with a SuperX EPR049 microwave bridge and a cylindrical TE011 ER 4122SHQE cavity in connection with an Oxford Instruments continuous flow cryostat. Measuring temperatures were achieved using liquid helium flow through an ITC 503 temperature controller (Oxford Instruments). Modulation frequency at 100 kHz was set in all measurements. Bruker Xepr software package was used for all data processing. The EPR simulation was performed using Bruker-Xsophe/XeprView software package (v.1.1.4). For the spin ½ system, only Zeeman interaction is included in the spin Hamiltonian operator. The simulated spectra were generated by matrix diagonalisation of anisotropic *g*-tensors for a randomly oriented spin system.

Protein film electrochemistry sample preparation

Samples for protein film electrochemistry were prepared under strictly anaerobic conditions by mixing reconstituted *Tam* HydS (50 µM) with sodium dithionite (1 mM, 20x excess) and $[2Fe]^{adt}$ (600 µM, 12x excess) in sodium phosphate buffer (100 mM, pH 6.8) and incubated in room temperature for 30 min. The activation was stopped by running the reaction through a PD-10 column (GE Healthcare) equilibrated with Tris-HCl buffer (100 mM, pH 8), 150 mM NaCl, 5% v/v glycerol and 2 mM sodium dithionite. The sample was concentrated using Amicon®Ultra 30 kDa cutoff centrifugal filters (Merck Millipore Ltd.) and aliquoted into airtight serum vials that were flash-frozen in liquid N_2 and stored at -80°C.

Protein film electrochemistry

Protein film electrochemistry experiments were carried out under strictly anaerobic conditions in an Ar atmosphere. The three-electrode system used comprised an isolated Ag/AgCl reference electrode (Fisher Scientific) held in a Luggin sidearm containing 0.1 M NaCl and a 0.5 mm platinum wire (Sigma Aldrich) as a counter electrode. All potentials were converted to the Standard Hydrogen Electrode (SHE) scale using the correction $E_{SHE} = E_{SCE} + 210$ mV at 25 °C. The working electrode was either a home-build pyrolytic graphite edge plane electrode (PGE) or glassy carbon electrode (GCE) rotated at 0–3000 rpm depending on the experiment. The PGEs working electrodes were made of a 2 mm diameter graphite rod embedded into a PEEK body. A tin rod attached to the graphite by a silver conductive epoxy adhesive (MG Chemicals) was used as an electrical lead to the electrodes. The GCEs used were purchased from Pine Research (E2M Fast Speed, 5 mm diameter). The glass cell used featured a water jacket for temperature control.

Four different procedures were tested for protein film preparation:

(A) The PGE electrode surface was abraded with P1200 sandpaper before being placed in a sonicator for approximately 1 min and then rinsed with purified water. An enzyme solution (5 µL of 2-5 µM in 10 mM HEPES buffer pH 7) was then pipetted onto the electrode surface and left for 5 min to adsorb before the excess of the solution was removed by pipette.

(B) The GCEs were successively polished with alumina powders (1.0, 0.3 and 0.05 µm) followed by ultrasonication in distilled water. The GCEs were functionalized with multi-wall carbon nanotubes as described earlier.[10] The MWCNTs-modified electrodes were used for enzyme immobilization as described in procedure (A).

(C) The PGEs were polished on P1200 sandpaper, sonicated and placed to a 0.2mM solution of 4-carboxybenzenediazonium in acetonitrile for 5 min. The electrodes were then washed with distilled water and ethanol. 1 µl of 36 mM N-(3-dimethylaminopropyl)-N'-ethylcarbodiimide (EDC) and 1µl of 18 mM 1N-hydroxysuccinimide (NHS) in water were painted on the electrode surface and let dry for 10 min; the electrode was then washed with water. 2 µl of 0.5 µM enzyme solution in 10 mM HEPES buffer pH 7 was dropped onto the electrode surface and let react for 5 min.[11]

(D) The PGEs were polished on P1200 sandpaper, sonicated and an enzyme solution (5 µL of 2-5 µM in 10 mM HEPES buffer pH 7) and polymyxin (5 µL of 0.2mg mL^{-1} in water) were then pipetted onto the electrode surface and left for 5-10 min to adsorb before the excess of the solution was removed by pipet.

Data analysis was performed using OriginPro 8 software. Buffer composition was a mixture of MES, CHES, HEPES, TAPS and sodium acetate, 5 mM each, with NaCl (0.1 M) as carrying electrolyte titrated with NaOH or HCl to the desired pH. Electrochemical data were acquired using an Eco/Chemie PGSTAT10 and the GPES software (Metrohm/Autolab). Data were analyzed using Origin 8 software.

Temperature study experiments on either proton reduction or hydrogen oxidation were completed with the same film at temperatures varying between 10 and 70°C. No significant protein film loss was observed during a set of experiments up to 60°C. The pH study experiments were performed with the same film at pHs between 5-9.

FTIR sample preparation

Samples for FTIR were prepared under strictly anaerobic conditions by mixing reconstituted *Tam* HydS (100 µM) with sodium dithionite (2 mM, 20x excess) and $[2Fe]^{adt}$ (500 µM, 5x excess) in sodium phosphate buffer (100 mM, pH 6.8) and incubated in room temperature for 1 h. The activation was stopped by running the reaction through a NAP-25 column (GE Healthcare) equilibrated with Tris-HCl buffer (10 mM, pH 8). The sample was concentrated using Amicon®Ultra 30 kDa cutoff centrifugal filters (Merck Millipore Ltd.) and aliquoted into airtight serum vials that were flash-frozen in liquid N_2 and stored at -80°C.

FTIR spectroscopy

FTIR spectroscopy was performed in ATR configuration on hydrated films of 1 µl isolated [FeFe]-hydrogenase (about 500 µM). Enzymes included *Tam* HydS, *Dd* HydAB, and *Cr* HydA1. All experiments were performed inside a Coylab glovebox (N_2 atmosphere with 1 – 2% H_2). Absorbance spectra were recorded on a Bruker Tensor 27 with a spectral resolution of 2 cm^{-1} and a varying number of interferometer scans at 80 MHz (time resolution 1 - 10 s). The spectrometer was equipped with a 3-reflections Si ATR unit (Smiths DuraSamplIR II). All gas-, light-, and potential titrations were performed according to established protocols.[12-14] For the temperature titrations, a stainless steel heat jacket was modified to fit the ATR crystal plate, which simultaneously served as a gas cell (see Fig. S2 below). A Julabo circulation pump was used to adjust the temperature of the heat jacket and ATR elements (20° - 40°C). A digital thermo couple was used to measure the temperature at the interface of reflection element and crystal plate. This conservative approach was chosen to compensate for the difference in heat conductance of the silicon ATR crystal (~1.3 W K^{-1} m^{-1}) and the stainless steel crystal plate (~15 W K^{-1} m^{-1}).

Supplementary tables and figures

Figure S1. SDS-PAGE of purified Tam HydS

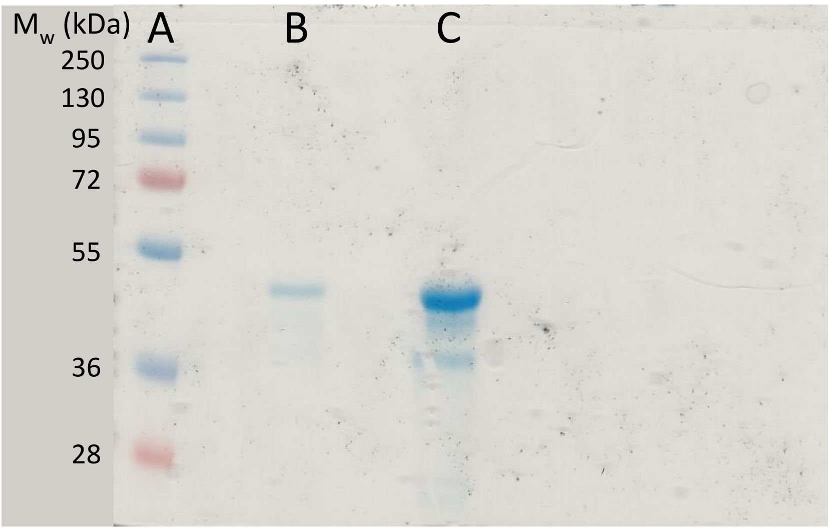

Figure S1. SDS-PAGE of purified *Tam* HydS. Lanes from left to right: Molecular weight marker (**A**); 6 µg (**B**) and 30 µg (**C**) of purified *Tam* HydS.

Figure S2. Schematic overview of the ATR-FTIR setup

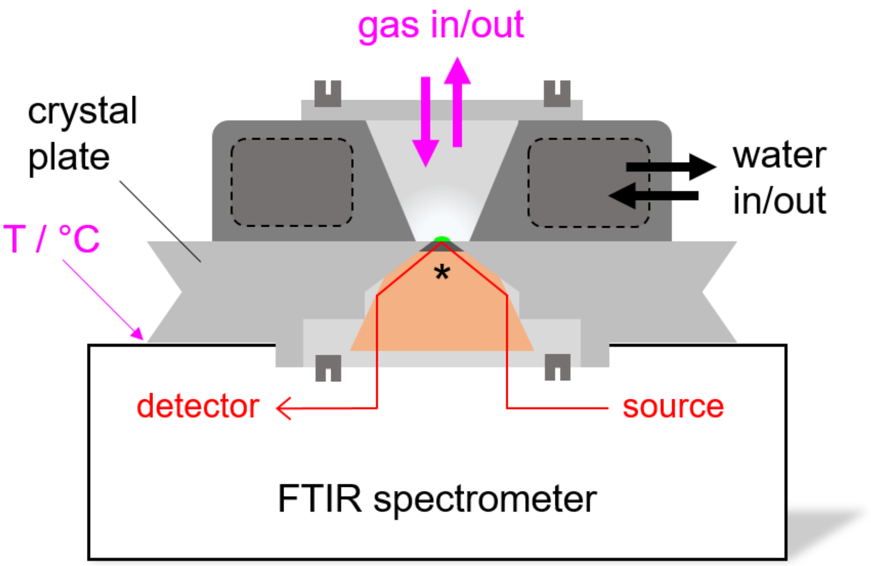

Figure S2. Schematic overview of the ATR-FTIR setup. The DuraDisc crystal plate is equipped with a stainless steel heat jacket, connected to a Julabo circulation pump that facilitates adjusting the temperature of the crystal plate. A thermo couple is attached between the crystal plate and the spectrometer surface. The atmosphere above the sample film (green) is sealed with an acrylic glass lid, equipped with an inlet and outlet for gas purging. The preparation of gas mixture is reported elsewhere.[13]

Figure S3. g-tensor assignment of [4Fe4S] clusters.

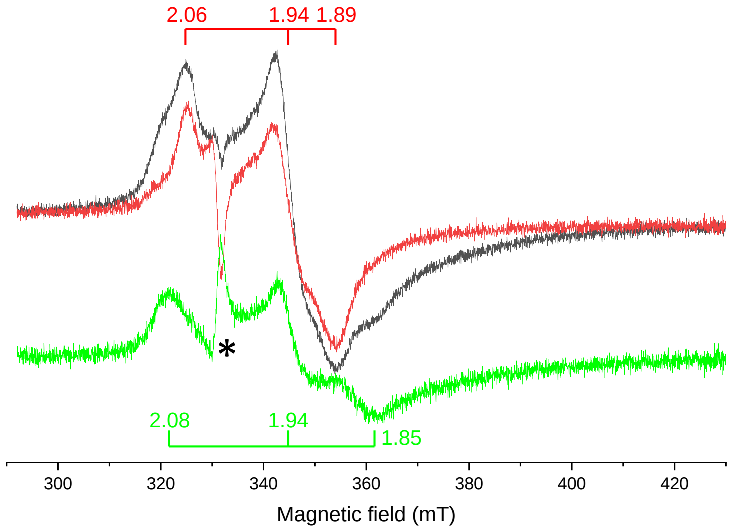

Figure S3 g-tensor assignment of [4Fe4S]-clusters. Comparing sodium dithionite (NaDT) reduced samples of *apo*- and *holo-Tam* HydS reveal the absence of one rhombic component in the latter. **Black** spectrum: NaDT reduced *apo-Tam* HydS; **Red** spectrum: NaDT reduced *holo-Tam* HydS; **Green** difference (= *apo* – *holo*) spectrum, representing the signal attributed to $[4Fe\text{-}4S]_H$. A sharp feature marked with * at g =2.03 is attributed to a small amount of H_{ox}-CO present in *holo-Tam* HydS. EPR settings: T 15 K; Modulation frequency 100 kHz, amplitude 10 G; Microwave frequency 9.4 GHz; power 100 µW. For power saturation data of *holo-Tam* HydS see Figure S4.

Figure S4. Power saturation study of the EPR signals of *holo-Tam* HydS.

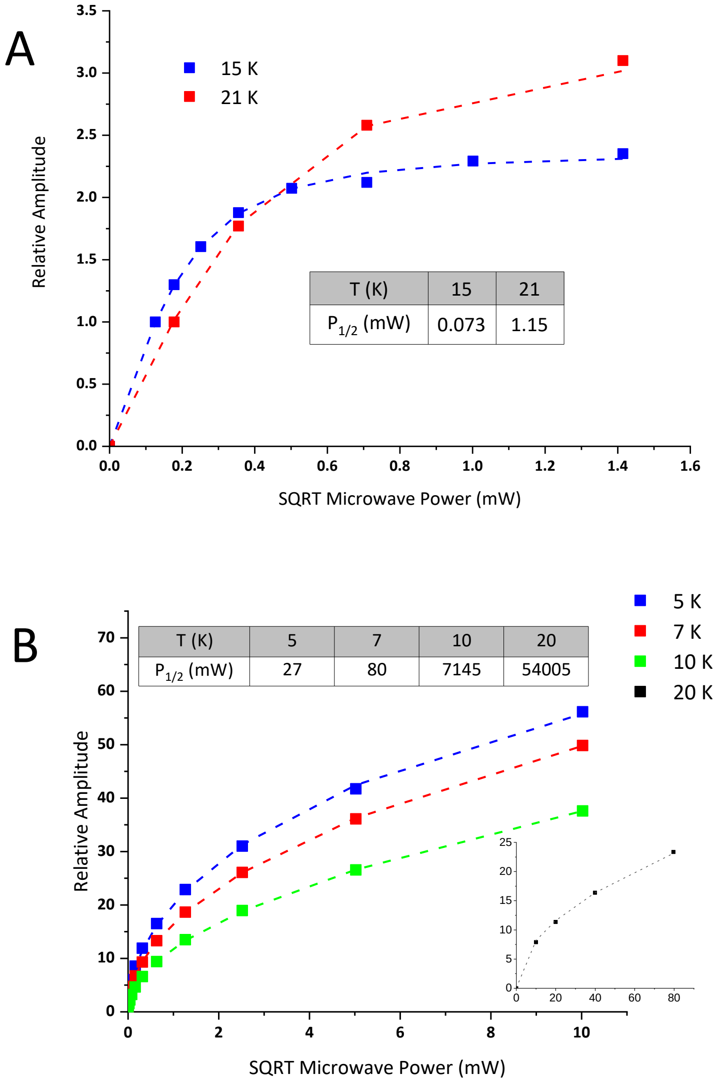

Figure S4. (**A**) Power saturation studies on the EPR signal from *holo-Tam* HydS matured with 2[Fe]^{adt}, at 15 K and 21 K. $P_{1/2}$ values resulted from data fitting (dashed lines) using the following equation: $I = I_0\sqrt{P}/\sqrt{1+P/P_{1/2}}$, are listed in the inset table. The power saturation plot is based on the peak intensities observed at g = 2.04 – 2.02, however, all spectra features followed a highly similar trend. (**B**) Power saturation studies on the EPR signal from NaDT reduced *holo-Tam* HydS, at 5 K, 7 K, 10 K and 20 K. $P_{1/2}$ values resulted from data fitting (dashed lines) using the following equation: $I = I_0\sqrt{P}/\sqrt{1+P/P_{1/2}}$, are listed in the inset table.

In agreement with previous reports, it was also observed in this work that the signals arising from the $[4Fe4S]^+$ clusters have pronounced spin anisotropy, resulting in much broader EPR spectra than those from the H-cluster, and the latter tends to suffer from microwave power saturation at much lower power than the $[4Fe4S]^+$ cluster(s) at equal temperature. This is illustrated in the obtained $P_{1/2}$ values (listed in the tables inserted in S4A and B) studied at varied temperature.

Guided by Figure S4 only the spectra retaining linearity were used for spin quantification. The linear region in graph **B** is noteworthy. The broad, seemingly “never saturating” $[4Fe4S]^+$ signals have a very narrow linear region, at least below 10 K, although the $P_{1/2}$ values are large. This clearly indicates that the spin diffusion mechanism largely contributes to the relaxation mechanism for the $[4Fe4S]^+$ cluster even at a temperature as low as 5 K. Nevertheless, for an accurate spin quantification, it is essential that the linearity hold. Thus, microwave power with sub-milliwatts were applied in spin quantifications, albeit at the expense of signal/noise quality.

Figure S5. Supporting EPR data on *holo-Tam* HydS (effect of pH and reductant; and simulations)

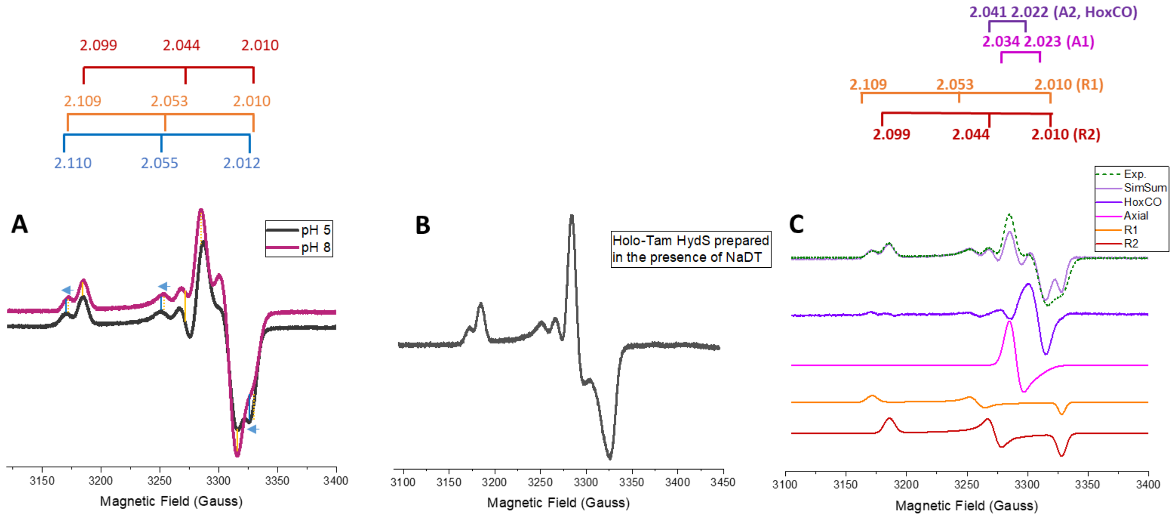

Figure S5. (**A**) The effect of pH on the EPR spectrum of *holo-Tam* HydS. Comparing spectra recorded of *holo-Tam* HydS samples prepared in pH 8 and pH 5 buffer allows g assignment for the two rhombic signals (assigned to H_{ox} and $H_{ox}H$)**.** Bordeaux spectrum: The rhombic signal (R1) with g = 2.109, 2.053 and 2.010 (yellow dashed bars) at pH 8 was downshifted ~ 2.5 – 3 Gauss (indicted with arrows) to the Black spectrum recorded at pH 5. The other rhombic signal (R2) with g = 2.099, 2.044 and 2.010 (yellow solid bars) was not affected by pH change. The peak positions of the axial signal with g = 2.041 and 2.022 assigned to H_{ox}-CO state was not affected by pH change, but the signal intensity decreased. (**B**) Effect of activation of *apo-Tam* HydS with $[2Fe]^{adt}$ in the presence of NaDT. Samples of *holo-Tam* HydS prepared in the presence of NaDT and then isolated via a desalting column showed a spectrum highly similar to N_2 flushed samples, indicated by the loss of H_{ox}-CO under these conditions. EPR settings: T 21 K; Modulation frequency 100 kHz, amplitude 10 G; Microwave frequency 9.4 GHz, power 16 µW. (**C**) Simulation assisted deconvolution of EPR spectrum obtained for *holo-Tam* HydS activated with $[2Fe]^{adt}$ and flushed with N_2 (Figure 4, spectrum B main manuscript). Top spectra is the overlay between the simulation (solid line) and the experimental data (dashed line). Apart from the H_{ox}-CO signal, three additional components contribute. Two rhombic spectra R1 and R2 and one axial signal. g-tensors are illustrated above. Based on the simulated spectra, double integration of the signals indicates that R2 accounts for 54.6%, R1, 25.2%, H_{ox}-CO signal, 7.8% and the axial A1 signal for 12.4%. This 2:1 ratio between R2 and R1 is in reasonable agreement with the ratios observed between H_{ox} and $H_{ox}H$ by FTIR at pH 8 (Figure S7), further supporting the assignment of R2 to H_{ox} and R1 to $H_{ox}H$.

Figure S6. FTIR spectroscopic characterization of pdt-*Tam* HydS

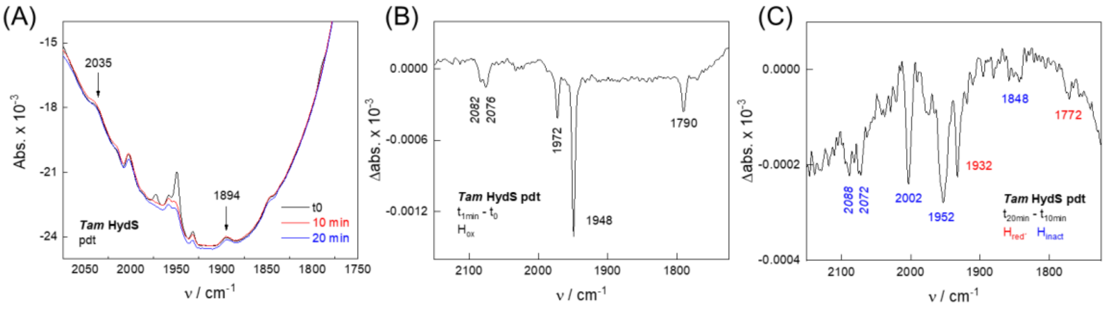

Figure S6. FTIR spectroscopic characterization of pdt-*Tam* HydS. **(A)** 1 µL pdt-*Tam* HydS solution (500 µM, pH 8) was used to form films for ATR FTIR spectroscopy. At room temperature and in the presence of N_2, the sample was found to reside in a mixture of redox states apparently dominated by H_{ox}. We observed no reactivity with H_2 or CO; however, in the presence of air, the cofactor slowly disintegrated. The untreated spectra show the CO/CN bands of the H-cluster before reaction with air (t_0) and 10 min and 20 min after. Bands at 2035 cm^{-1} and 1894 cm^{-1} in the absolute spectrum of pdt-*Tam* HydS are tentatively assigned to H_{red}. This state has never been reported for pdt cofactor variants of [FeFe] hydrogenase before. The corresponding bands did not decrease in the presence of air (compare (B) and (C)), which impeded a closer characterization of the parent H-cluster state. **(B)** $t_{1\,min} – t_0$ difference spectrum. In the early phase of oxidative damage, the data show that H_{ox} reacted first with O_2. Its loss facilitated isolating the IR signature of H_{ox}. Unlike *holo*-*Tam* HydS prepared with $[2Fe]^{adt}$, we did not observe $H_{ox}H$ in pdt-*Tam* HydS. **(C)** $t_{20\,min} – t_{10\,min}$ difference spectrum. In the late phase of oxidative damage, $H_{red´}$ and a H_{inact}-like state were identified. We did not observe any trace of H_{ox}-CO, neither before or during the reaction with O_2.

Figure S7. Accumulation of $H_{ox}H$ at low pH in the presence of reductant.

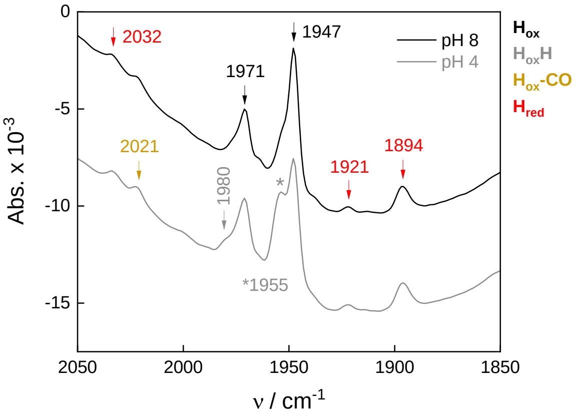

Figure S7. Accumulation of $H_{ox}H$ at low pH in the presence of reductant. The spectra depict the composition of H-cluster states after 24 h under N_2 at pH 8 (black trace) and pH 4 (gray trace). The aerosol buffer was enriched with 2 mM sodium dithionite. Besides a minor contribution of H_{red} and H_{ox}-CO (marker bands at 1894 cm^{-1} and 2021 cm^{-1}) the spectra comprised of ~65% H_{ox} and ~25% $H_{ox}H$ at pH 8 and ~50% H_{ox} and ~40% $H_{ox}H$ at pH 4. In contrast to other previously studied [FeFe]-hydrogenases,[13] increasing the dithionite concentration in the aerosol buffer to 10 mM did not facilitate a complete conversion into $H_{ox}H$.

Figure S8. ATR-FTIR spectro-electrochemical study of the H_{ox}/H_{red} interconversion.

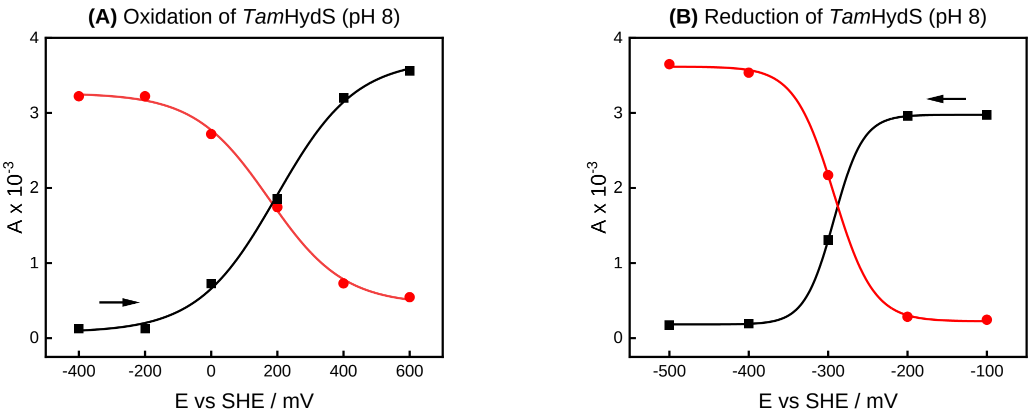

Figure S8. 1µL *Tam* HydS solution (~500 µM, pH 8) was injected into a gold mesh that had been placed onto the ATR silicon crystal before. No electron mediators were used. Following established protocols[12], an electrochemical cell was attached to the ATR unit. Then, the beaker was filled with electrolyte solution (10 mM "mixed buffer" pH 8 with 100 mM KCl) and brought into contact with the protein film (the hydration process was monitored by FTIR spectroscopy). The electrolyte was carefully purged with N_2 throughout the experiment to remove traces of H_2. The gold mesh was connected to the working electrode, a platinum wire was used as counter electrode, and an Ag/AgCl element was exploited as reference electrode.

At the open circuit potential, the enzyme adopted the reduced state, H_{red}. **(A)** In the first step, we increased the potential from -400 mV vs SHE by 200 mV for 30 min each (until equilibrium conditions) and plotted the absorbance at 1947 cm^{-1} (H_{ox}, black squares) and 1896 cm^{-1} (H_{red}, red circles). At +600 mV vs SHE, >90% of the film was oxidized, suggesting a midpoint potential of E_m^{ox} ≈ +200 mV (sigmoidal fits, solid black/red traces). The film showed <10% H_{ox}H that appeared to behave similar to H_{ox}. **(B)** In the second step, we lowered the potential by 100 mV for 30 min each. Surprisingly, no changes were observed until -200 mV vs SHE when the enzyme converted back into H_{red}. At -500 mV vs SHE, ~100% of the film was reduced, suggesting a midpoint potential of E_m^{red} ≈ -300 mV vs SHE (sigmoidal fits, solid black/red traces).

Electrochemical reduction is well compatible with single electron transfer, as expected for the transition of H_{ox} into H_{red}. At -300 mV, E_m^{red} is comparatively anodic, reflecting the nature of H_{red} as metastable 'resting state' in *Tam* HydS (see main text). For comparison, the H_{ox}/H_{red} midpoint potential of the sensory hydrogenase *Tm* HydS is equally high[15] whereas prototypical hydrogenases were reported with E_m = -390 mV (*Dd* HydAB)[16] or E_m = -380 mV (*Cr* HydA1)[17]. It should be noted that the observed potential of the Hox/Hred interconversion is pH dependent due to the protonation of the [2Fe] subsite, and in the case of Dd HydAB also dependent on the oxidation states of the F-clusters. For a more in-depth discussion see e.g. ref. [18]. In *Tam* HydS, electrochemical oxidation occurs at ~500 mV more positive potentials and displayed a broad profile incompatible with single electron transfer.[19]

Figure S9. Formation of H_{air} and H_{ox}-CO under oxidizing and reducing conditions.

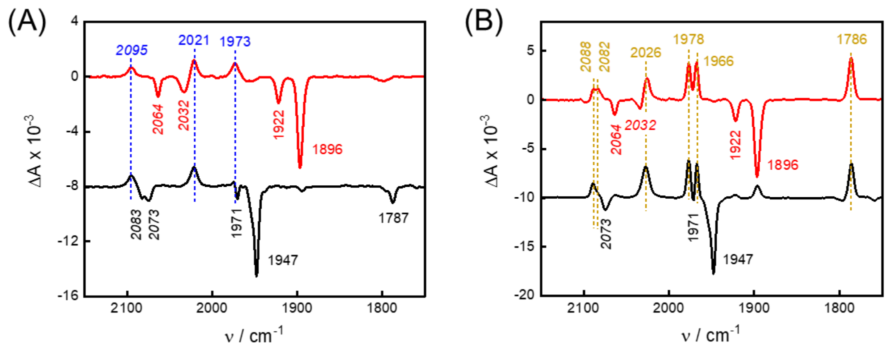

Figure S9. Formation of H_{air} and H_{ox}-CO under oxidizing and reducing conditions. (**A**) Films of *Tam* HydS (pH 8) were prepared under 1% H_2 and 100% N_2 to accumulate H_{red} and H_{ox}/H_{ox}H, respectively. When the carrier gas was mixed with 50% air (≈10% O_2), immediate conversion into H_{air} was observed (positive bands), notably independent of the parent state (red trace, H_{air} – H_{red}; black trace, H_{air} – H_{ox}/H_{ox}H). We did not observe H_{air}-red as product of oxidative damage of the reduced H-cluster. (**B**) Films of *Tam* HydS were prepared under 1% H_2 and 100% N_2 to accumulate H_{red} and H_{ox}/H_{ox}H, respectively. When the carrier gas was mixed with 10% CO, conversion into H_{ox}–CO was observed (positive bands), notably independent of the parent state (red trace, H_{ox}-CO – H_{red}; black trace, H_{ox}-CO – H_{ox}/H_{ox}H). We did not observe $H_{red'}$-CO as product of CO inhibition of the reduced H-cluster.

Figure S10. Full kinetic traces for the reaction with H_2 and/or CO in *Tam* HydS and *Dd* HydAB.

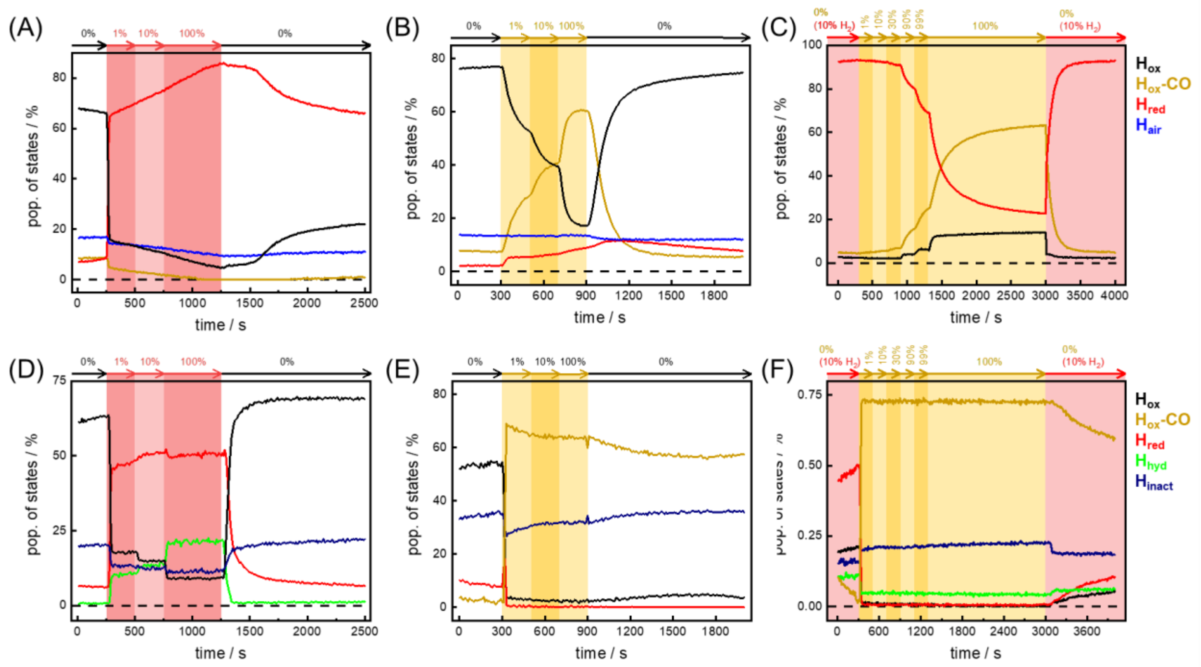

Figure S10. Full kinetic traces for the reaction with H_2 and/or CO in *Tam* HydS and *Dd* HydAB. All experiments at pH 8, 24°C. **(A)** H_2 oxidation of *Tam* HydS in the presence of N_2. The fraction of H_{air} (blue trace) barely reacts to H_2. Note the gradual decrease of H_{ox}-CO (brown trace). Overall, the reaction with H_2 is instantaneous; however, auto-oxidation (0% H_2) is extremely slow. **(B)** CO inhibition of *Tam* HydS in the presence of N_2. The fraction of H_{air} (blue trace) does not react to CO. Note the gradual increase of H_{red} (red trace). Overall, the reaction with CO is slow and incomplete. In the absence of CO, H_{ox} (black trace) quickly recovers. **(C)** CO inhibition of *Tam* HydS in the presence of 10% H_2. Due to competition of H_2 and CO for the same coordination site at the H-cluster, significant CO inhibition was noted only for CO concentrations larger than 90%. The equilibrium under 100% CO gas was the same as in panel (B). Note the gradual increase of H_{ox} (black trace). Overall, the reaction with CO is slow and incomplete. We did not observe H_{air} in this sample. **(D)** H_2 oxidation of *Dd* HydAB in the presence of N_2. The fraction of H_{inact} (dark blue trace) is partly activated under H_2 but recover in the absence of H_2. Overall, the reaction with H_2 is instantaneous; equally, auto-oxidation quickly recovers H_{ox} (black trace). Unlike *Tam* HydS, co-accumulation of H_{red} and H_{hyd} was observed (red and green traces, respectively). **(E)** CO inhibition of *Dd* HydAB in the presence of N_2. The fraction of H_{inact} (dark blue trace) barely reacted to CO. Overall, the reaction with CO is instantaneous; however, in the absence of CO, H_{ox} (black trace) barely recovers. **(F)** CO inhibition of *Dd* HydAB in the presence of 10% H_2. Due to the pronounced sensitivity of the H-cluster to CO, no significant differences to panel (E) were observed (different to *Tam* HydS, we did not detect significant competition of H_2 and CO). The fraction of H_{inact} (dark blue trace) barely reacted to CO. Note the small yet significant fraction of H_{hyd} under CO.

Figure S11. Full kinetic traces for the evaluation of the temperature effect in *Tam* HydS and *Dd* HydAB.

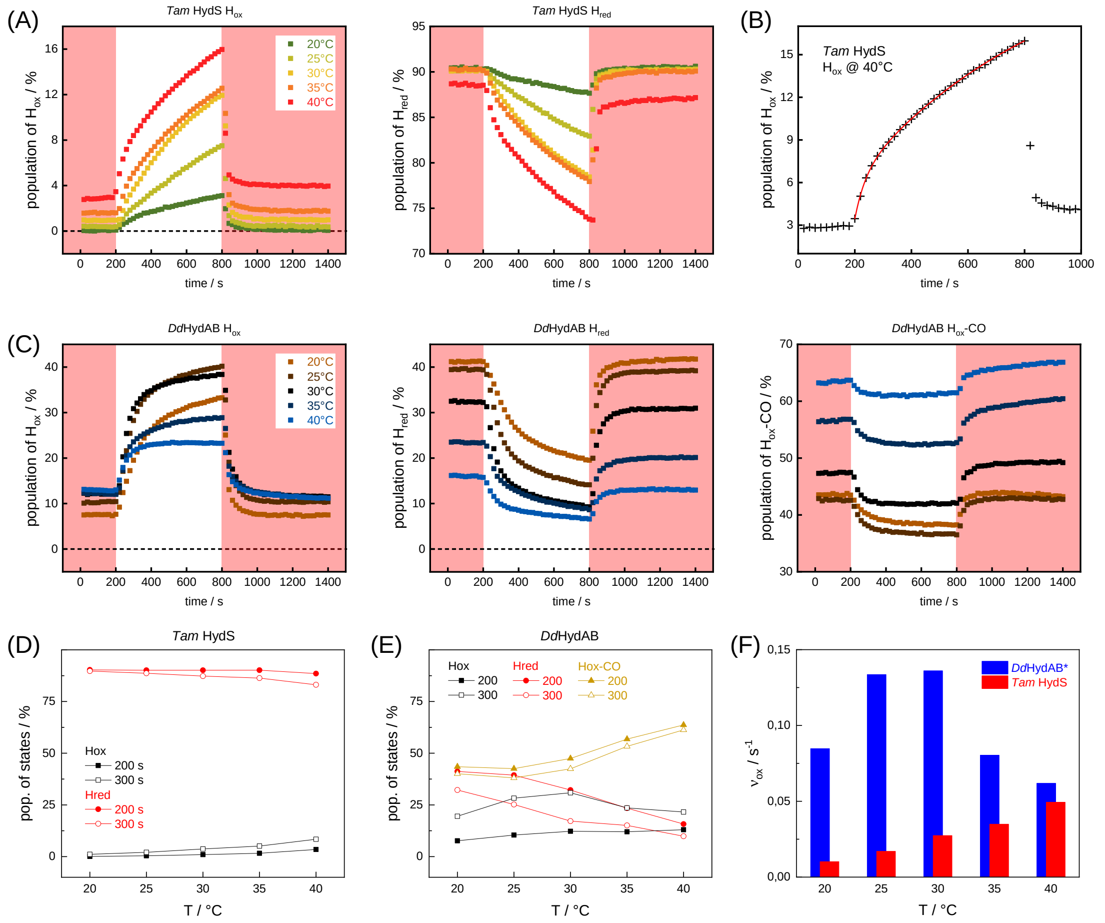

Figure S11. Full kinetic traces for the evaluation of the temperature effect in *Tam* HydS and *Dd* HydAB. **(A)** Auto-oxidation of *Tam* HydS in the absence of H_2 for five different temperature steps (20°C – 40°C). The film was changed from 1% H_2 to 100% N_2 (≈ 0% H_2) and back to 1% H_2. Changes of H_{ox} (left) and H_{red} (right) over time are depicted. The accumulation of H_{ox}-CO at elevated temperature in *Tam* HydS is barely significant. **(B)** The increase of H_{ox} (here at 40°C) fitted with bi-exponential kinetics with a 'fast' and a 'slow' component, suggesting multiple pathways for this reaction. **(C)** Auto-oxidation of *Dd* HydAB in the absence of H_2 for five different temperature steps (20°C – 40°C). The film was changed from 1% H_2 to 100% N_2 (≈ 0% H_2) and back to 1% H_2. Changes of H_{ox} (left), H_{red} (middle), and H_{ox}-CO (right) over time are depicted. The accumulation of H_{ox}-CO was found to increase with temperature. Moreover, H_{ox}-CO is very stable in *Dd* HydAB (compare Figure S9) and not easily reactivated. **(D)** The plot shows the percentage of H_{ox} and H_{red} at t = 200s and t = 300 s (i.e., before and 100 s after the atmosphere was changed from 1% H_2 to N_2) between 20°C – 40°C. In *Tam* HydS, H_{red} clearly dominates the composition of redox states. The percentage of H_{ox}-CO is insignificant (data not shown). **(E)** The plot shows the percentage of H_{ox}, H_{red}, and H_{ox}-CO at t = 200s and t = 300 s between 20°C – 40°C. In *Dd* HydAB, H_{red} is the least dominant state at t = 300 s, as expected for a prototypical [FeFe]-hydrogenase in the absence of H_2. Note the high percentage of H_{ox}-CO that increases with temperature. **(F)** Based on the increase of H_{ox} in the absence of H_2, the temperature dependence of the auto-oxidation activity in *Tam* HydS (red bars) and *Dd* HydAB (blue bars) was evaluated. For this, we calculated:

$$v_{ox}(T) = \frac{([H_{ox}]_{300}^{T} - [H_{ox}]_{200}^{T})}{100\ s}$$

In order to compensate for the CO-inhibited fraction of *Dd* HydAB, we calculated:

$$v_{ox}(T) = \frac{([H_{ox}]_{300}^{T} + [H_{ox}CO]_{300}^{T})}{100\ s} - \frac{([H_{ox}]_{200}^{T} + [H_{ox}CO]_{200}^{T})}{100\ s}$$

The diagram illustrates a continous increase of v_{ox} with temperature in *Tam* HydS. Between 20°C and 30°C, v_{ox} is approximately ten times higher in *Dd* HydAB. At temperatures > 30°C, the activity drops significantly, which is in marked difference to *Tam* HydS.

Figure S12. Accumulation of super-oxidized states in *Dd* HydAB under O_2.

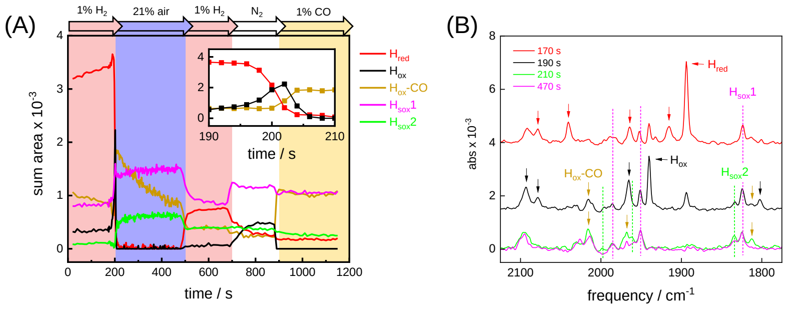

Figure S12. Accumulation of super-oxidized states in *Dd* HydAB under O_2. (A) The enzyme was reduced in the presence of 1 % H_2 to accumulate H_{red}. There is a smaller contribution of oxidized H-cluster states (H_{ox}, H_{ox}-CO) and a “super-oxidized” state $H_{sox}1$. When the gas phase was switched to air (t=200 s), H_{red} quickly dropped whereas H_{ox} transiently peaked and vanished from the spectrum (see inset). The steep increase in H_{ox}-CO suggested H-cluster disintegration following oxidative damage. Additionally, the super-oxidized state $H_{sox}1$ increased in the presence of air, and a blue-shifted state $H_{sox}2$ was identified. We did not observe H_{air} (i.e., as in *Tam* HydS) or H_{ox}-O_2 (i.e., as in *Cr HydA1*). $H_{sox}1$ and $H_{sox}2$ were partly re-activated under 1% H_2. In the last step, the enzyme was subjected to 1% CO, which had no effect on $H_{sox}1$ and $H_{sox}2$ but converted both H_{ox} and H_{red} into H_{ox}-CO. In summary, these data demonstrate why a direct comparison between *Tam* HydS and *Dd* HydAB is not possible. **(B)** Spectra at different time points. Directly before the reaction with O_2 (t = 170 s), the spectrum is dominated by H_{red} (and a small fraction of $H_{sox}1$ is observed). At t = 190 s, H_{red} is transiently converted into H_{ox}. Spectra at t = 210 s and t = 470 s facilitate identification of the CO band signature for $H_{sox}1$ and $H_{sox}2$ (Table S1). These states are likely related to H_{inact} and H_{trans} but show an overall red-shifted IR signature.[20]

Table S1. IR signature of H-cluster states observed in *Dd* HydAB (CO-only)

Table S1. IR signature of H-cluster states observed in *Dd* HydAB (CO-only). All data given in cm^{-1}.				
**Hox-CO**	2015	1968	1962	1812
**Hred**	1964	1915	1894	
**Hox**		1964	1940	1802
**Hsox1**		1984	1951	1824
**Hsox2**		1998	1960	1834

Figure S13. EPR-spectra in the g ≈ 2 region of as prepared and reduced H_{air}

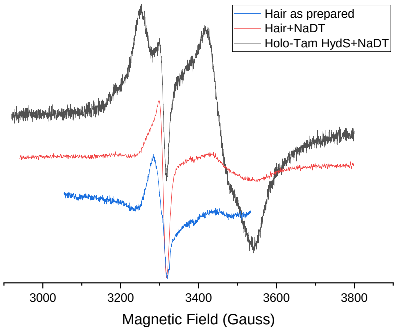

Figure S13. EPR spectra in the g ≈ 2 region of as prepared and reduced H_{air}. The H_{air} state was prepared by exposing a solution of *Tam* HydS (50 μM) to air for 10 minutes, after which the sample was made anaerobic and either flash-frozen in liquid nitrogen (H_{air} as-prepared) or reduced by 20 equivalents of NaDT (1 mM) for 10 minutes and then flash-frozen (H_{air} reduced). Spectra recorded at 17 K of H_{air} as-prepared (blue spectrum) and H_{air} reduced (red spectrum) are rescaled to be comparable with the *holo-Tam* HydS reduced NaDT (black spectrum). A narrow feature at g = 2.02 observed in H_{air} as-prepared is attributed to a $[3Fe4S]^+$ species. This feature was essentially unchanged by the addition of NaDT, instead a new broad but weak feature appeared at g =1.89, recognized as belonging to $[4Fe\text{-}4S]^+$ (see the field position in comparison with the black spectrum. In terms of spin quantity, both H_{air} as-prepared H_{air} reduced are practically EPR silent (spin concentration 1 – 2 μM).

Figure S14. ^{13}CO isotope editing and spectro-electrochemical characterization of H_{air}-ox and H_{air}-red.

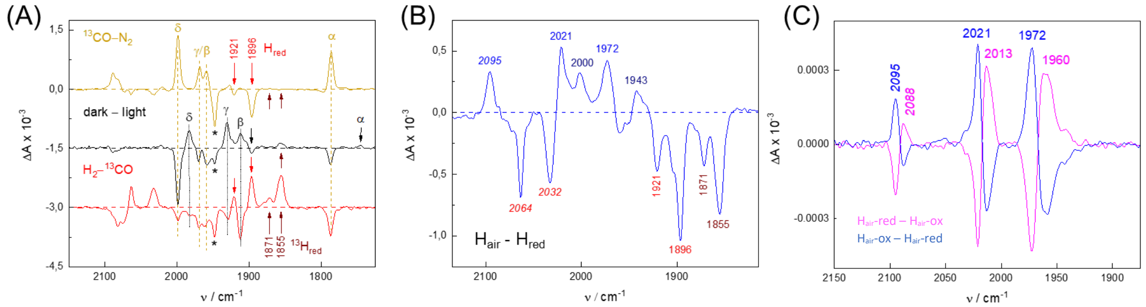

Figure S14. ^{13}CO isotope editing and spectro-electrochemical characterization of H_{air}-ox and H_{air}-red. (**A**) A film of *Tam* HydS was partly oxidized and reacted with 3 atm ^{13}CO following established protocols.[14] The dark yellow difference spectrum shows the conversion of H_{red} (as indicated by red arrows) and H_{ox} (*1948 cm^{-1}) into H_{ox}-^{13}CO. White light irradiation in the presence of ^{13}CO facilitates isotope scrambling and produces an inhibited H-cluster with three terminal ^{13}CO ligands (H_{ox}-(^{13}CO)$_3$), black spectrum). Surprisingly the μCO ligand was barely exchanged and only a very small μ^{13}CO band at 1744 cm^{-1} was observed (α). Reactivating the H-cluster in the presence of 1 atm H_2 (red spectrum) demonstrated a 40% editing efficiency which reflects the instability of H_{ox}-CO in *Tam* HydS (compare Figure S9). Bands at 1871 and 1855 cm^{-1} are assigned to isotopically labelled $^{13}H_{red}$ (dark red). The respective shifts are 50 and 40 cm^{-1}, indicative of Fe_p-^{13}CO and Fe_d-^{13}CO. (**B**) In the second step, the labelled enzyme was subjected to 1 atm air. The difference spectrum depicts the conversion of H_{red} (red) and $^{13}H_{red}$ (dark red) into H_{air} (light blue) and $^{13}H_{air}$ (dark blue). For the latter, the respective shifts are 21 and 29 cm^{-1}, indicative of vibrational coupling between ^{12}CO and ^{13}CO bound to a single iron ion. A similar coupling pattern was observed for the [Fe](CO)$_2$CN site of 'Complex B' as bound to the [FeFe]-hydrogenase maturase HydG.[21] This "damped" shift can be explained by the loss of the distal iron upon reaction with O_2. The un-labelled μCO ligand (compare panel (A)) turns into a terminal ligand at the proximal iron ion, which remains associated with the [4Fe-4S] cluster. In summary, H_{air} can be described as a mono iron H-cluster complex with two terminal CO ligands and a single CN ligand (the high frequency ligand at 2095 cm^{-1} was not affected by ^{13}CO editing protocol, which supports the assignment to cyanide). The IR signatures of all isotopomers observed in this study are compiled in Table S2. (**C**) The presence of the [4Fe-4S] cluster was verified by ATR FTIR spectro-electrochemistry on O_2-deactivated films of *Tam* HydS. Following established protocols,[12] potential jump experiments between -150 mV and -550 mV vs SHE demonstrate the existence of a reduced form of H_{air}, denoted H_{air}-red. The small downshifts suggest reduction of the [4Fe-4S] cluster, not unlike the spectral differences between H_{ox} and $H_{red'}$.[13] The conversion was found to be reversible (magenta trace, reduction; blue trace, oxidation). Whether or not H_{air} still carries the adt ligand is matter of speculation; unfortunately, H_{air} was not observed in *Tam* HydS pdt (compare Figure S6). The small but discernable difference between adt and pdt are well established in the case of the full H-cluster,[22] and could have been useful to conclude on the presence of absence of a dithiolate ligand in H_{air}. Given the octahedral nature of the iron ions in the intact H-cluster, the mono Fe species in H_{air} may form six bonds - one with the bridging cysteine, one with cyanide and two with the carbonyls. Due to the fact that H_{air} shows highly limited reactivity towards both H_2 and CO (compare Figure S9), the remaining two coordination sites are arguably occupied. Therefore, we suggest the presence of azadithiolate in H_{air} (Figure S13).

Table S2. Isotopomers generated in this study.

<table>
<tr><td></td><td colspan="2" rowspan="2">CN</td><td colspan="12">CO</td></tr>
<tr><td></td><td colspan="3">δ</td><td colspan="3">γ</td><td colspan="3">β</td><td colspan="3">α</td></tr>
<tr><td>H_{ox}-CO</td><td>2088</td><td>2082</td><td colspan="3">2026</td><td colspan="3">1978</td><td colspan="3">1966</td><td colspan="3">1786</td></tr>
<tr><td>H_{ox}-^{13}CO</td><td>2088</td><td>2082</td><td colspan="3">1998</td><td colspan="3">1968</td><td colspan="3">1958</td><td colspan="3">1786</td></tr>
<tr><td>H_{ox}-(^{13}CO)$_3$</td><td>2088</td><td>2082</td><td colspan="3">1982</td><td colspan="3">1930</td><td colspan="3">1912</td><td colspan="3">1786</td></tr>
<tr><td>H_{ox}-(^{13}CO)$_4$</td><td>2088</td><td>2082</td><td colspan="3">1982</td><td colspan="3">1930</td><td colspan="3">1912</td><td colspan="3">1744</td></tr>
<tr><td>H_{red}</td><td>2064</td><td>2032</td><td colspan="4">n.d.</td><td colspan="4">1921</td><td colspan="4">1896</td></tr>
<tr><td>$^{13}H_{red}$</td><td>2064</td><td>2032</td><td colspan="4">n.d.</td><td colspan="4">1871</td><td colspan="4">1855</td></tr>
<tr><td>H_{air}</td><td colspan="2">2095</td><td colspan="6">2021</td><td colspan="6">1972</td></tr>
<tr><td>$^{13}H_{air}$</td><td colspan="2">2095</td><td colspan="6">2000</td><td colspan="6">1943</td></tr>
</table>

Figure S15. Suggested geometry of H_{air}.

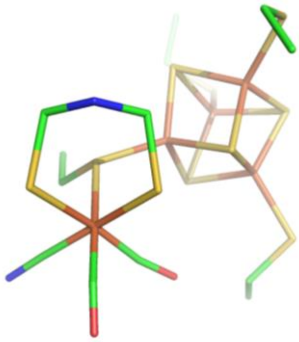

Figure S15. Suggested geometry of H_{air}. Model based on pdb coordinates 4XDC.[23] The hydrogen bonding between protein fold and Fe_p-CN is likely to stabilize the depicted ligand geometry.[24]

Figure S16. Optimization of the immobilization protocol in PFE.

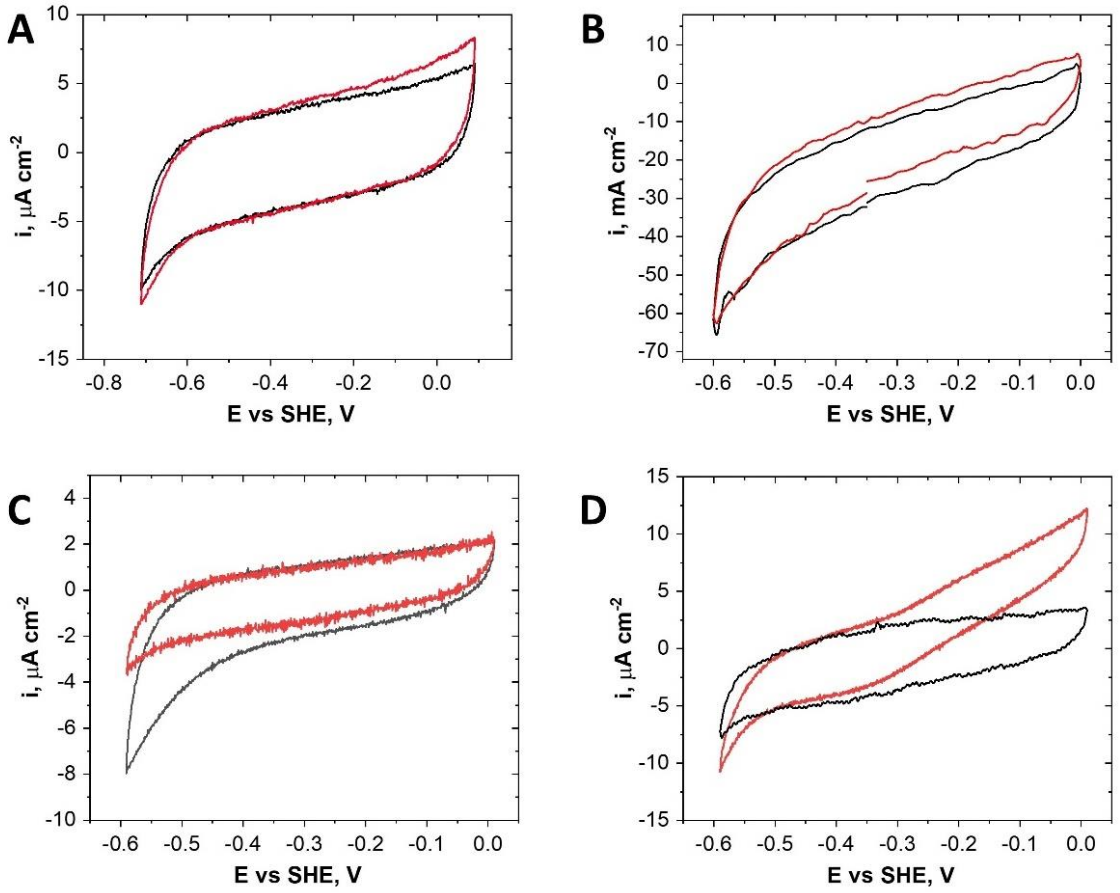

Figure S16. Optimization of the immobilization protocol in PFE. CVs of electrodes prior to enzyme immobilization (black) and with *holo-Tam* HydS under 1 atm H_2 (red). (**A**) Absorption on a freshly polished pyrolytic graphite electrode (PGE). (**B**) Absorption on a glassy carbon electrode (GCE) modified with multiwalled carbon nanotubes (MWCNTs). (**C**) Covalent attachment via EDC/NHS chemistry to a PGE modified with 4-carboxybenzendiazonium and (**D**) Absorption on a freshly polished PGE in the presence of polymyxin B sulfate. The scan rate is 10 mV s^{-1}, the rotation rate is 3000 rpm, pH 7, 30°C.

Figure S17. Influence of H_2 partial pressure on currents observed in PFE.

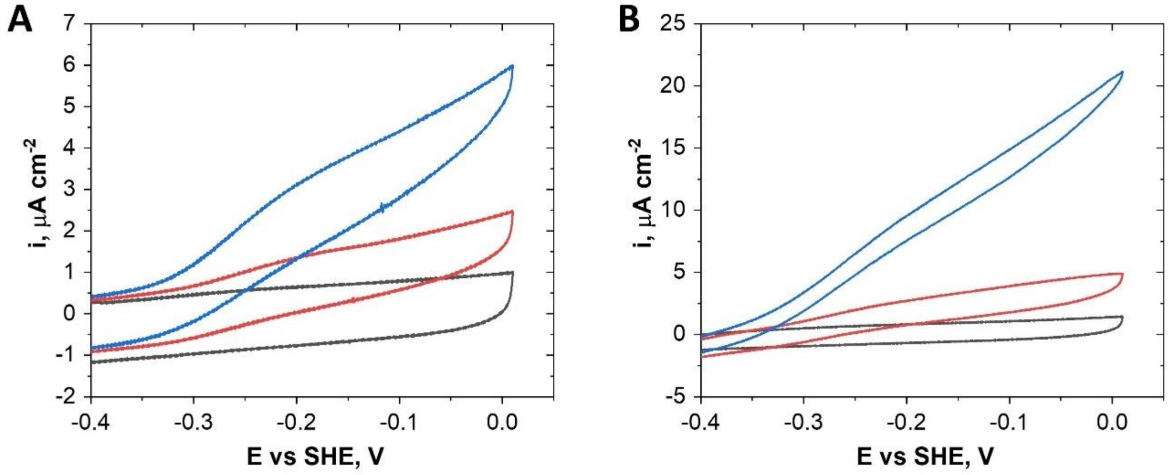

Figure S17. Influence of H_2 partial pressure on currents observed in PFE. Examples of CVs obtained at rotating disk PGEs modified with *holo-Tam* HydS in a pH 7 buffer solution under Ar (black), 7.5% H_2 (red) and 100% H_2 (blue) at (**A**) 30°C and (**B**) 60°C. The scan rate is 2 mV s^{-1}, the rotation rate is 3000 rpm.

Figure S18. Influence of pH on currents observed in PFE.

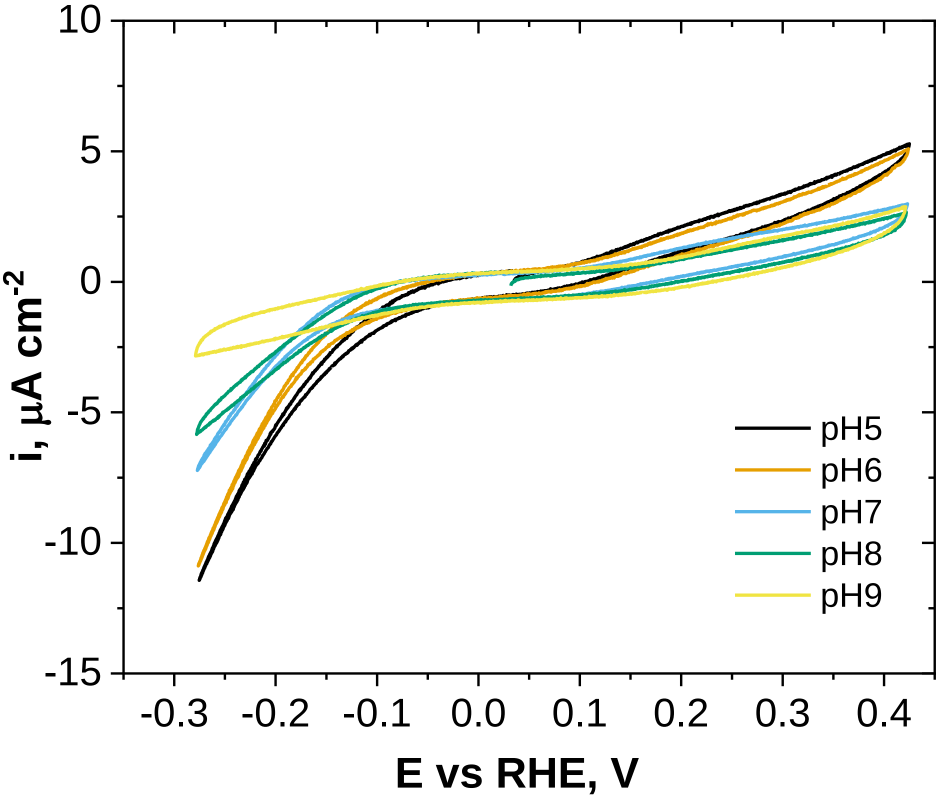

Figure S18. Influence of pH on currents observed in PFE. CVs obtained at a rotating disk PGE modified with *holo-Tam* HydS under 1 atm H_2 at various pHs from 5 to 9 at 30°C. The potential is normalized to the reversible hydrogen electrode (RHE). The scan rate is 2 mV s^{-1}, the rotation rate is 3000 rpm. The onset potential for H^+ reduction decreases with lowering pH, with overpotential required for H^+ reduction being close to zero at pH 5. The onset potential for H_2 oxidation also decreases with pH with observable switching to smaller overpotentials when going from pHs 9-7 to pH 6-5.

Figure S19. Stability of protein film in PFE.

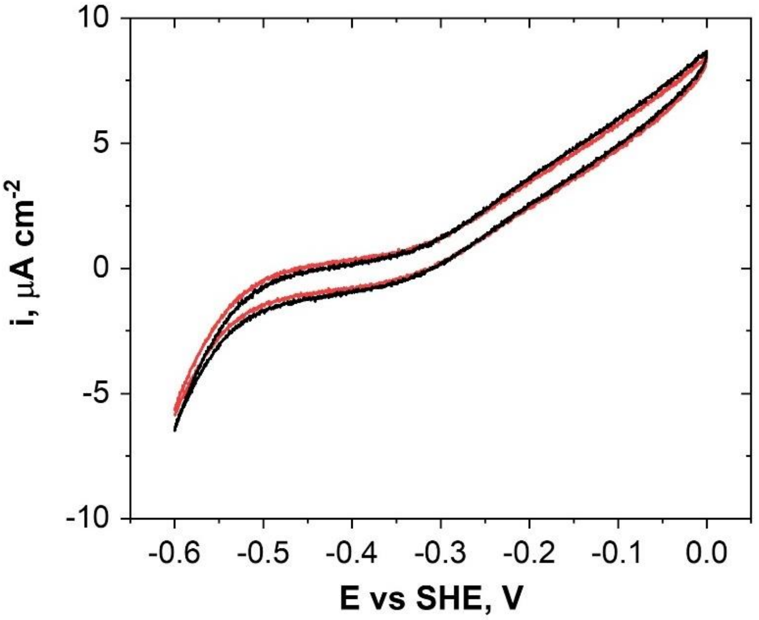

Figure S19. Stability of a protein film in PFE. CVs obtained at a rotating disk PGE modified with *holo-Tam* HydS under 1 atm H_2 in pH 7 solution at 30°C before the temperature dependence experiment (black) and after the experiment stopped at 60°C (red). The time of the experiment is ~4 hours, during which the solution with the electrode was heated from 10°C to 60°C and CVs were recorded for each 10°C increase in temperature at 3000 rpm. The scan rate is 2 mV s^{-1}, the rotation rate is 3000 rpm.

Figure S20. Reduction currents observed for H_{air}

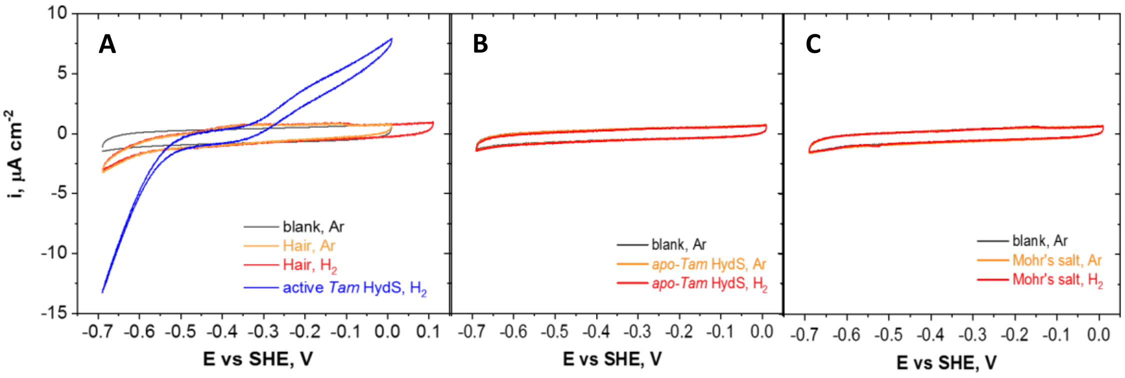

Figure S20. Reduction currents observed for H_{air}. (**A**) CVs obtained at rotating disk PGEs modified with the active *holo-Tam* HydS under 1atm H_2 (blue), the enzyme in H_{air} state under 1 atm H_2 (red), and the enzyme in H_{air} state under Ar (orange). Background CV is shown in black. The scan rate is 2 mV s^{-1}, the rotation rate is 3000 rpm, pH 7, 30°C. Once exposed to air to form H_{air}, the enzyme lost capacity for H_2 oxidation, but retained H^+ reduction capacity, albeit with an increase in over-potential. (**B**) The protein in *apo*-form does not show any catalytic activity. (**C**) The electrode modified with 5 µl of 5 µM $(NH_4)_2Fe(SO_4)_2·6H_2O$ (Mohr's salt) does not show any catalytic activity.

Table S3. K_M^{H2} calculated using estimated i_{lim}.

(δi/δE)×T of the high driving force part of the oxidation part of the cyclic voltammograms at various concentrations of H_2, pH 7.		
**H_2 concentration, %**	(δi/δE)×T	
	30°C	60°C
**0**	$(1.5 \pm 0.3)\times10^{-5}$	$(3.9 \pm 0.6)\times10^{-5}$
**7.5**	$(5.0 \pm 0.6)\times10^{-5}$	$(21.4\pm 0.9)\times10^{-5}$
**100**	$(12.4\pm 0.3)\times10^{-5}$	$(63 \pm 11)\times10^{-5}$
**K_M^{H2}, mM**	0.09 ± 0.02	0.10± 0.03

Figure S21. Active site of *Tam* HydS homology model

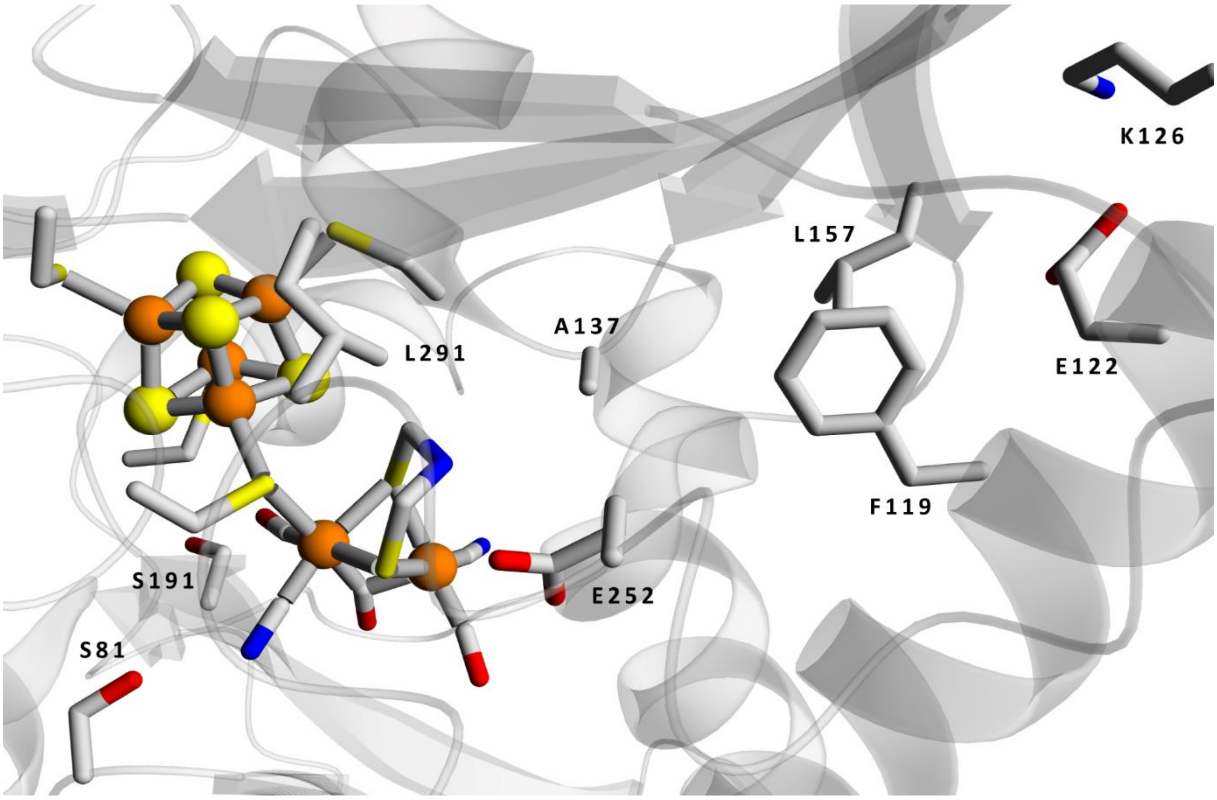

Figure S21. Structural view of the active site and proton transfer pathway of a truncated *Tam* HydS homology model missing the C-terminal domain. The H-cluster is taken from an aligned structure of *Cp*I (PDB ID: 4XDC) since the homology model did not contain $[2Fe]^{adt}$.

References

1. H. Li and T. B. Rauchfuss, *J. Am. Chem. Soc.*, 2002, **124**, 726-7.
2. R. Zaffaroni, T. B. Rauchfuss, D. L. Gray, L. De Gioia and G. Zampella, *J. Am. Chem. Soc.*, 2012, **134**, 19260-9.
3. A. Le Cloirec, S. C. Davies, D. J. Evans, D. L. Hughes, C. J. Pickett, S. P. Best and S. Borg, *Chem. Commun.*, 1999, 2285-2286.
4. M. Schmidt, S. M. Contakes and T. B. Rauchfuss, *J. Am. Chem. Soc.*, 1999, **121**, 9736-9737.
5. E. J. Lyon, I. P. Georgakaki, J. H. Reibenspies and M. Y. Darensbourg, *Angew. Chem. Int. Ed.*, 1999, **38**, 3178-3180.
6. L. S. Mészáros, B. Németh, C. Esmieu, P. Ceccaldi and G. Berggren, *Angew. Chem. Int. Ed.*, 2018, **57**, 2596-2599.
7. M. M. Bradford, *Anal. Biochem.*, 1976, **72**, 248-254.
8. W. W. Fish, in *Methods in enzymology*, Academic Press, 1988, vol. 158, pp. 357-364.
9. H. Land and M. S. Humble, in *Protein Engineering. Methods in Molecular Biology*, eds. U. T. Bornscheuer and M. Höhne, Humana Press, New York, NY, 2018, vol. 1685, pp. 43-67.
10. H. Land, P. Ceccaldi, L. S. Mészáros, M. Lorenzi, H. J. Redman, M. Senger, S. T. Stripp and G. Berggren, *Chem. Sci.*, 2019, **10**, 9941-9948.
11. C. Baffert, K. Sybirna, P. Ezanno, T. Lautier, V. Hajj, I. Meynial-Salles, P. Soucaille, H. Bottin and C. Léger, *Anal. Chem.*, 2012, **84**, 7999-8005.
12. M. Senger, K. Laun, F. Wittkamp, J. Duan, M. Haumann, T. Happe, M. Winkler, U.-P. Apfel and S. T. Stripp, *Angew. Chem. Int. Ed.*, 2017, **56**, 16503-16506.
13. M. Senger, S. Mebs, J. Duan, O. Shulenina, K. Laun, L. Kertess, F. Wittkamp, U.-P. Apfel, T. Happe, M. Winkler, M. Haumann and S. T. Stripp, *Phys. Chem. Chem. Phys.*, 2018, **20**, 3128-3140.
14. M. Senger, S. Mebs, J. Duan, F. Wittkamp, U.-P. Apfel, J. Heberle, M. Haumann and S. T. Stripp, *Proc. Natl. Acad. Sci. U.S.A.*, 2016, **113**, 8454-8459.
15. N. Chongdar, J. A. Birrell, K. Pawlak, C. Sommer, E. J. Reijerse, O. Rüdiger, W. Lubitz and H. Ogata, *J. Am. Chem. Soc.*, 2018, **140**, 1057-1068.
16. W. Roseboom, A. L. De Lacey, V. M. Fernandez, E. C. Hatchikian and S. P. J. Albracht, *J. Biol. Inorg. Chem.*, 2006, **11**, 102-118.
17. C. Sommer, A. Adamska-Venkatesh, K. Pawlak, J. A. Birrell, O. Rüdiger, E. J. Reijerse and W. Lubitz, *J. Am. Chem. Soc.*, 2017, **139**, 1440-1443.
18. V. Engelbrecht, P. Rodriguez-Macia, J. Esselborn, A. Sawyer, A. Hemschemeier, O. Rudiger, W. Lubitz, M. Winkler and T. Happe, *Biochim. Biophys. Acta, Bioenerg.*, 2017, **1858**, 771-778.
19. M. Senger, V. Eichmann, K. Laun, J. Duan, F. Wittkamp, G. Knör, U.-P. Apfel, T. Happe, M. Winkler, J. Heberle and S. T. Stripp, *J. Am. Chem. Soc.*, 2019, **141**, 17394-17403.
20. P. Rodríguez-Maciá, E. J. Reijerse, M. van Gastel, S. DeBeer, W. Lubitz, O. Rüdiger and J. A. Birrell, *J. Am. Chem. Soc.*, 2018, **140**, 9346-9350.
21. J. M. Kuchenreuther, W. K. Myers, D. L. M. Suess, T. A. Stich, V. Pelmenschikov, S. A. Shiigi, S. P. Cramer, J. R. Swartz, R. D. Britt and S. J. George, *Science*, 2014, **343**, 424-427.
22. A. Adamska-Venkatesh, D. Krawietz, J. Siebel, K. Weber, T. Happe, E. Reijerse and W. Lubitz, *J. Am. Chem. Soc.*, 2014, **136**, 11339-11346.
23. J. Esselborn, N. Muraki, K. Klein, V. Engelbrecht, N. Metzler-Nolte, U. P. Apfel, E. Hofmann, G. Kurisu and T. Happe, *Chem. Sci.*, 2016, **7**, 959-968.
24. O. Lampret, A. Adamska-Venkatesh, H. Konegger, F. Wittkamp, U.-P. Apfel, E. J. Reijerse, W. Lubitz, O. Rüdiger, T. Happe and M. Winkler, *J. Am. Chem. Soc.*, 2017, **139**, 18222-18230.
